# Exposure levels of animal allergens, endotoxin, and β-(1,3)-glucan on a university campus of veterinary medicine

**DOI:** 10.1371/journal.pone.0288522

**Published:** 2023-07-13

**Authors:** Eva Zahradnik, Ingrid Sander, Anne Lotz, Verena Liebers, Ingrid Thullner, Sabine Tacke, Monika Raulf

**Affiliations:** 1 Institute for Prevention and Occupational Medicine of the German Social Accident Insurance, Institute of the Ruhr-Universität Bochum (IPA), Bochum, Germany; 2 Unfallkasse Hessen (UKH), Frankfurt am Main, Germany; 3 Veterinary Medicine Clinic, Justus-Liebig-University Gießen, Gießen, Germany; VIT University, INDIA

## Abstract

**Objectives:**

The study aimed to determine the allergen, endotoxin and β-(1,3)-glucan concentrations at various areas on a university campus of veterinary medicine.

**Methods:**

Dust samples were collected four times a year for three years using electrostatic dust collectors (EDC) at 25 different locations on a campus of veterinary medicine and in laboratories of inorganic chemistry as a control area representing animal-free environment. Major animal allergens from dog, cat, horse, cattle and mouse, domestic mite (DM) allergens, and β-(1,3)-glucan were measured using enzyme immunoassays and endotoxin using the limulus amoebocyte lysate (LAL) assay. Seasonal, annual and local influences on exposure levels were analyzed using Bayesian mixed models.

**Results:**

With the exception of mouse allergens, all other determinants were found in almost all locations on the campus and in the control area, but in up to 10.000-fold variable concentrations. By far the highest levels of feline, canine, equine and bovine allergens were detected in buildings where the respective species were examined. The highest levels of mouse and DM allergens, β-(1,3)-glucan and endotoxin occurred together and were associated with locations where large animals were present. In buildings without animals, allergen levels were considerably lower but still elevated at several locations compared to the control area, especially for dog and horse allergens, and β-(1,3)-glucan. Significant seasonal effects were observed for dog, cat, horse and DM allergens, and β-(1,3)-glucan. Variations between years were less apparent than between seasons (except for β-(1,3)-glucan).

**Conclusions:**

The strongest influencing factor on the concentration of mammalian allergens was the presence of the corresponding animal at the collection site. Seasonal influence on allergen concentrations was observed, while the overall exposure remained constant over the years. At locations with horses, elevated levels of mite allergens, endotoxin, and β-(1,3)-glucan can be expected, probably due to passive transfer from stable environment.

## Introduction

Occupational handling of animals, e.g. in veterinary clinics, veterinary practices or animal stables, is expected to result in high exposures to various components of bioaerosols including mammalian allergens, mite allergens and microbial components such as endotoxin and β-(1,3)-glucan [1–6]. In particular, unavoidable exposure to animal allergens carries the risk of sensitization and allergic symptoms [7]. Several major allergens from diverse animal species are well characterized with regard to their molecular structure and immunogenicity [8]. In fur-bearing mammals, the major allergens (recognized by specific IgE antibodies in >50% of patients allergic to the allergen source) are considered marker proteins for the proof of sensitization as well as for allergen detection in dust samples, i.e. their quantification represents a good approximation of actual allergen exposure. In contrast, the allergenic potential of mites is usually distributed among several individual allergens in parallel, with different personal relevance for the allergy sufferers and strongly variable content within the allergen source. Several mite species are known worldwide to cause allergic rhinitis and asthma, each with its own specific allergens [9]. Here, the quantification of only one single allergen might not be adequate as a proxy of allergen exposure. A useful method to detect diverse mite allergens is the “domestic mite assay” developed by Sander et al. [10]. Due to the broad spectrum of detection of different species and its high sensitivity, this assay has often been used as a screening tool for mite allergen exposure [2, 11–13]. The determination of endotoxins (a part of the outer membrane of Gram-negative bacteria) and β-(1,3)-glucans (a cell-wall component of molds) is widely used in occupational and environmental medicine to characterize bioaerosols. Both agents are not allergenic but can stimulate the human immune system in different ways (inflammatory, pyrogenic, irritant or toxic effects) and therefore represent a possible risk factor for the development of respiratory diseases [14].

The most common occupational hazards for veterinarians are injuries of a physical nature. These include injuries from animals (bites, scratches and hits), needle sticks and musculoskeletal disorders (caused by lifting animals or working in improper postures) [15]. Nevertheless, allergies represent also a considerable proportion of professional risks [16–21]. Furthermore, a Dutch study in veterinary students has shown an increased prevalence of allergic symptoms with elevated years of veterinary study, suggesting that the contact with animals, in particular farm animals, is a risk factor for the development of symptoms [22]. Further studies are needed to determine or estimate exposure levels and to find out a possible dose-response relationship between bioaerosol exposure and health outcomes. Generally, it is not possible to determine the exposure levels of biological agents precisely, as they can vary extremely in time and space depending on the activity and working materials, and also depend on external influencing factors (e.g. temperature, humidity). Therefore, a comprehensive exposure assessment for specific location requires repeated measurements to account for temporal variations in airborne levels of different bio-contaminants [23].

The objective of our study was to characterize exposure levels during the education of veterinary students. In order to obtain as representative a picture as possible of exposure in the different areas of the veterinary medicine settling airborne dust measurements were performed for three years during each season at various locations on a university campus of veterinary medicine. This included indoor measurement and quantification of major allergens from cat (Fel d 1), dog (Can f 1), horse (Equ c 1), cattle (Bos d 2), mouse (Mus m 1) and domestic mite (DM) allergens. Furthermore, endotoxin and (1–3)-β-glucan as markers of bacterial and mold load were determined. These exposure levels were compared with those of laboratories of inorganic chemistry as a control representing animal-free environment. Additionally, seasonal and annual variations of exposure levels were analyzed.

## Materials and methods

### Exposure assessment

The study was conducted at the Faculty of Veterinary Medicine of the Justus Liebig University Giessen, Hesse, Germany. The electrostatic dust collector (EDC) with two dust-binding cloths (Techmed-Textil-Service-GmbH, Dipperz, Germany) was used to collect settled airborne dust. The sampling procedure, documentation, and extraction were the same as previously described for our study in veterinary practices [2]. Dust sampling took place from 2014 until 2016 four times a year during each season (February, May, August, November) at 25 different locations in 15 buildings. These locations were chosen because these are the places most frequently visited by the students during their training period. In total, 96 dust samples were collected in 2014 and in 2015, and 91 samples in 2016.

The control measurements were carried out in the laboratories (n = 8) of the Institute of Inorganic Chemistry on the Natural Sciences Campus of the Justus Liebig University Giessen, which is spatially separated from the Veterinary Medicine Campus (distance approx. 1000 m). This area had similar conditions in terms of occupancy, use, climate conditions, and annual cycles, differing only in the presence of animals. This allowed the appropriate rating for the extent of exposure to bio-contaminants on the veterinary campus. Dust sampling took place for two years during each season in the location. In total, 32 dust samples were collected in the control area.

This study was part of the project AllergoVet which was approved by the Ethics Committee of the Ruhr University Bochum in Germany (registration number: 4810–13).

### Quantification of allergens, β-(1,3)-glucan and endotoxin

Enzyme immunoassays (EIA) or fluorescence enzyme immunoassays (FEIA) in sandwich format were used to quantify allergens of various animal species and β-(1,3)-glucan. The assays used are based on either monoclonal or polyclonal antibodies purchased commercially from Indoor (Indoor Biotechnologies, Charlottesville, VA. USA) or generated at the IPA by immunization of rabbits. The chromogenic substrate 2.2’-azino-bis-(3-ethylbenzothiazoline-6-sulfonic acid) (ABST, Sigma, Steinheim, Germany) was used for EIAs and the fluorogenic substrate QuantaBlu (ThermoScientific, Rockford, IL, USA) for FEIAs. An overview of the assay characteristics including the reference for the exact protocol is given in Table 1.

**Table 1:** Summary of enzyme immunoassays.

Species	Allergen/target agent	Calibration standard	Antibodies	Limit of detection	Assay variant	Provider	Reference
**Cat**	Fel d 1	nFel d 1	monoclonal	0.01 ng/ml	FEIA	Indoor	[24]
**Dog**	Can f 1	nCan f 1	monoclonal	0.01 ng/ml	FEIA	Indoor	[24]
**Mouse**	Mus m 1	nMus m 1	polyclonal	0.01 ng/ml	FEIA	Indoor	[24]
**Cattle**	Bos d 2	rBos d 2	monoclonal	0.02 ng/ml	EIA	Indoor	[25]
**Horse**	Equ c 1	nEqu c 1	polyclonal	0.01 ng/ml	FEIA	IPA	[26]
**Domestic mite**	diverse mite proteins	*Dermatophagoides farinae* extract	polyclonal	0.05 ng/ml	FEIA	IPA	[10]
**Moulds**	β-(1,3)-Glucan	carboxymethylated (CM)-curdlan	monoclonal	0.4 ng/ml	EIA	IPA	[27]

n: natural, r: recombinant, EIA: enzyme immunoassay, FEIA: fluorescence enzyme immunoassay, IPA: Institute for Prevention and Occupational Medicine of the German Social Accident Insurance

Endotoxin was determined using a kinetic chromogenic limulus amoebocyte lysate (LAL) assay Endosafe® Endochrome-K™ (Charles River, Sulzfeld, Germany) according to the manufacturer’s instructions. Briefly, lyophilised *E*. *coli* control standard endotoxin (CSE) was dissolved in pyrogen-free water and reconstituted to a concentration of 50 endotoxin units (EU)/ml. Sample extracts were thawed and diluted with pyrogen-free water 1:10 before measurement. 100 μl of the sample were incubated for 10 min at 20°C in a 96-well microplate (Falcon 3072, Becton Dickinson, Heidelberg, Germany). The LAL reagent (100 μl) was rapidly added to the samples and kinetics were recorded at 405 nm and 37°C using a temperature-controlled microplate reader (SpectraMax 340PC and software Softmax Pro 5.4.6, Molecular Devices, Sunnyvale, USA). For each measurement, a fresh standard curve in the range from 0.005 to 50 EU/ml was prepared. For the calculation of endotoxin concentrations a log-log fit of the standard curve was used. As positive controls, all samples were spiked with CSE (5 EU/ml). The values were accepted when the spike recovery was between 50% and 200%; otherwise the measurement was repeated. Pyrogen-free water served as negative control. All measurements were done in duplicate [28].

All exposure levels were expressed as ng/m^2^ for allergens and β-(1–3)-glucan or EU/m^2^ for endotoxin. The limit of detections (LODs) for EDC samples were 7.2 ng/m^2^ for Fel d 1, Can f 1, Equ c 1 and Mus m 1, 14.4 ng/m^2^ for Bos d 2 and 35.9 ng/m^2^ for DM. Endotoxin and β-(1,3)-glucan could be measured in all EDC samples.

### Statistical analysis

The descriptive statistics (geometric means with geometric standard deviations), Spearman correlation matrix and graphs were made with GraphPad Prism version 9.3.1 (GraphPad Software Inc., La Jolla, CA, USA). Values below the lower limit of detection (LOD) were replaced with 2/3 LOD for descriptive statistics. Statistical analyses were calculated using software OpenBUGS version 3.2.3 rev 1012 (Bayesian inference Using Gibbs Sampling) [29], R Statistical Software version 3.6.3 [30] and the R packages: bayestestR version 0.12.1 [31], plotMCMC version 2.0.1 [32], and R2WinBUGS version 2.1–21 [33].

The concentrations of the bio-contaminants were log-transformed and analyzed using mixed Bayesian models to determine 1) differences between each location and the control area, and 2) differences between seasons and years. Each analyte was analyzed in a separate model. Mus m 1 was not analyzed using mixed models due to very high percentage of samples below the detection limit (80%). A Bayesian analysis was chosen to address the hierarchical structure of the data due to repeated measurements, to address censored variables due to observations below a limit of detection, and to address missing values. Additionally, the Bayesian models allow to inspect generated parameters of interest looking at their posterior distribution.

Results of the Bayesian models were described by characterizing the posterior distribution of parameters of interest with measures of centrality (point estimate, calculated by the median of the posterior) and dispersion (95% credible interval, calculated by quantiles of the posterior as equal tailed interval, ETI). With caution, these can be considered the counterparts to the coefficient point-estimates and confidence intervals of the frequentist framework. Additionally, Bayesian hypothesis tests were conducted looking at a combination of the indices of effect existence (pd: Probabiliy of Direction) and significance (full ROPE: percentage of the posterior in the Region of Practical Equivalence) [34]. The pd is an index of effect existence representing the certainty with which an effect goes in a particular direction. It is strongly correlated with the frequentist p-value. The percentage in full ROPE is an index of significance informing whether a parameter is related or not to a non-negligible change. Table 2 provides some guidance for readers non-familiar with Bayesian statistics on how to interpret the two indices [35].

**Table 2:** Interpretation helper for effects existence and significance.

Index of existence			Index of significance	
Probability of direction (pd)	p-value equivalent	Interpretation	Full ROPE [% in ROPE]	Interpretation
**pd ≤ 95%**	≈ p > 0.1	uncertain	> 99%	negligible
**pd > 95%**	≈ p < 0.1	possibly existing	> 97.5%	probably negligible
**pd > 97%**	≈ p < 0.05	likely existing	≤ 97.5% - ≥ 2.5%	undecided significance
**pd > 99%**	≈ p < 0.01	probably existing	< 2.5%	probably significant
**pd > 99.9%**	≈ p < 0.001	certainly existing	< 1%	significant

To address the first research question (differences between each location and the control area), linear models of the log-transformed exposure as dependent variable were analyzed with location and season as independent variables. Broad, uninformative priors were used for all parameters. To address the second research question (differences between seasons and years), mixed linear models of the log-transformed exposure as dependent variable were analyzed with season and year as fixed effects and location as random effect. Observations from the control location were not included in this analysis. Again, uninformative priors were used for all parameters. Further details on the statistical models can be found in S1 Appendix. Results of the Bayesian hypothesis tests were presented in tables and figures by different numbers of asterisks with the following categorization

***: pd>99.9% (certainly existing) & <1% in ROPE (significant)

**: pd>99.9% (certainly existing) & <2.5% in ROPE (probably significant) **OR**

**: pd>99% (probably existing) & <1% in ROPE (significant)

*: pd>99% (probably existing) & <2.5% in ROPE (probably significant)

## Results

In total, 283 samples from the veterinary medicine campus were analyzed for all allergens and β-(1,3)-glucan, and 277 samples were tested for endotoxin. Can f 1 was detected in 87%, Fel d 1 in 73%, Equ c 1 in 98%, Bos d 2 in 83%, DM allergens in 62.5% and Mus m 1 in only 20% of the samples. Endotoxin and β-(1,3)-glucan were measurable in all samples. The distribution of the concentrations of all analytes sorted by location is shown in Table 3 (results of the Bayesian model described by point estimates and 95% credible interval). This table also provides information on whether the corresponding location differs significantly from the control area (indicated by asterisks). Detailed results of the Bayesian hypothesis tests can be found in S1 Table. The descriptive statistics (geometric means, geometric standard deviations and range) are presented in S2 Table.

**Table 3:** Animal allergen, endotoxin and β-(1,3)-glucan levels at different locations of the veterinary medicine campus.

Location						Fel d 1 (ng/m^2^)					Can f 1 (ng/m^2^)						Equ c 1 (ng/m^2^)							Bos d 2 (ng/m^2^)					
No	Building	Room		N		ND	PE		95% CI	BHT	ND	PE	95% CI		BHT		ND	PE		95% CI		BHT		ND	PE		95% CI		BHT
1	Small Animal Clinic, Internal Medicine	Seminar room		11		0	**269**		**134–540**	***	0	**1082**	**582–2020**		***		0	**769**		**327–1804**		***		1	32		17–61		ns
2	Small Animal Clinic, Surgery	Ultrasound room		12		0	**606**		**311–1182**	***	0	**3723**	**2058–6763**		***		0	**5006**		**2218–11352**		***		2	49		26–90		ns
3		Anesthesia room		12		0	**3057**		**1567–5956**	***	0	**12281**	**6771–22342**		***		0	**2298**		**1015–5190**		***		2	**54**		**29–100**		*
4		X-ray room		12		0	**83**		**43–163**	***	0	**1056**	**582–1907**		***		0	**13288**		**5883–30083**		***		0	**119**		**65–218**		***
5		Inpatient ward		8		1	**32**		**14–73**	*	0	**413**	**200–855**		***		0	66		25–181		ns		4	11		4.8–25		ns
6		Exhibit collection		12		0	25		13–48	ns	0	**276**	**152–501**		***		0	**704**		**312–1592**		***		2	28		15–52		ns
7	Equine Clinic, Surgery	Examination room		9		0	28		13–61	ns	0	**103**	**52–205**		***		0	**1112490**		**433303–2856378**		***		0	**329**		**164–662**		***
8	Equine Clinic, Internal Medicine	Corridor		12		1	**32**		**16–62**	***	0	**296**	**163–539**		***		0	**30757**		**13602–69616**		***		1	**117**		**63–214**		***
9	Ruminant Clinic	Lecture hall		12		5	14		6.7–28	ns	1	45	24–81		ns		0	**1215**		**534–2747**		***		0	**718**		**394–1316**		***
10	Clinic for Obstetrics	Lecture hall		12		5	16		7.6–32	ns	1	**109**	**59–198**		***		0	**5253**		**2331–11844**		***		0	**1929**		**1057–3531**		***
11		Locker room		10		1	**98**		**47–204**	***	0	**386**	**202–738**		***		0	**21971**		**9001–53923**		***		0	**15917**		**8207–30924**		***
12	Surgery lecture hall	Foyer		11		3	**36**		**17–74**	***	0	**291**	**157–542**		***		0	**1635**		**697–3814**		***		1	**55**		**29–105**		*
13	Institute of Anatomy	Practice room		12		6	5.4		2.4–11	ns	5	13	6.8–25		ns		0	74		33–167		ns		5	18		9.2–34		ns
14		Microscopy room		12		5	10		4.7–21	ns	3	27	14–50		ns		1	140		62–319		ns		4	24		12–44		ns
15		Lecture hall		10		3	23		11–50	ns	2	45	23–87		ns		0	**306**		**125–747**		*		4	22		11–43		ns
16		Locker room		11		3	**71**		**35–146**	***	3	**106**	**56–200**		***		1	**600**		**254–1419**		***		2	45		24–85		ns
17	Institute of Physiology	Lecture hall		12		5	14		6.6–28	ns	2	**79**	**43–145**		***		0	**245**		**108–554**		*		3	32		17–60		ns
18		Practice room		12		4	11		5.4–23	ns	2	**67**	**37–122**		***		0	**237**		**105–539**		*		1	**72**		**39–132**		***
19	Institute of Food Science	Practice room		11		4	12		5.5–25	ns	4	24	12–46		ns		1	103		44–242		ns		1	43		23–81		ns
20	Institute of Pathology	Practice room		12		8	4.7		2.0–10	ns	6	9.1	4.6–18		ns		0	96		43–218		ns		1	49		26–89		ns
21	Institute of Hygiene	Lecture hall		12		6	8.0		3.7–17	ns	2	21	11–38		ns		3	85		36–194		ns		4	33		17–62		ns
22		Practice room		12		3	15		7.3–29	ns	3	29	16–53		ns		0	172		76–389		ns		4	29		16–55		ns
23	Deanery	Office		12		7	4.0		1.7–8.7	ns	0	**149**	**83–271**		***		0	171		76–387		ns		5	21		11–40		ns
24	Learning Center	Computer room		10		1	23		11–48	ns	1	38	20–74		ns		0	**463**		**190–1129**		***		1	44		22–85		ns
25	Faculty council	Office		12		6	7.7		3.5–16	ns	1	**52**	**28–95**		*		0	**262**		**116–591**		*		1	32		17–58		ns
**CA**	Institute for Inorganic Chemistry	Laboratories		32		14	9.9		6.2–15		7	20	13–29				5	71		43–119				8	22		15–32		
**Location**					**Mus m 1 (ng/m^2^)**						**Domestic mite (ng/m^2^)**						**β-(1,3)-glucan (ng/m^2^)**							**Endotoxin (EU/m^2^)**					
**No**	**Building**		**Room**	**N**	**ND**		**GM**	**Range**			**ND**	**PE**		**95% CI**		**BHT**	**ND**		**PE**		**95% CI**		**BHT**	**N**		**ND**	**PE**	**95% CI**	**BHT**
1	Small Animal Clinic, Internal Medicine		Seminar room	11	10		5.4	<LOD-12.2			0	73		31–172		ns	-		**3565**		**1717–7339**		***	11		-	**362**	**192–686**	***
2	Small Animal Clinic, Surgery		Ultrasound room	12	7		8.0	<LOD-76.8			0	**142**		**62–323**		**	-		**7125**		**3526–14289**		***	12		-	**1193**	**651–2199**	***
3			Anesthesia room	12	10		5.6	<LOD-10.8			0	**198**		**87–451**		***	-		**6491**		**3231–12972**		***	11		-	**1315**	**693–2493**	***
4			X-ray room	12	7		8.5	<LOD-61.0			0	**134**		**59–307**		**	-		**10180**		**5064–20401**		***	12		-	**1912**	**1037–3519**	***
5			Inpatient ward	8	8		5.0	<LOD-<LOD			7	4,6		0,6–21		ns	-		**1869**		**798–4404**		*	8		-	59	28–125	ns
6			Exhibit collection	12	12		5.0	<LOD-<LOD			7	19		7,1–50		ns	-		**2451**		**1224–4922**		***	12		-	**184**	**100–339**	***
7	Equine Clinic, Surgery		Examination room	9	0		100	37.3–379			0	**3310**		**1288–8564**		***	-		**347403**		**154932–775658**		***	9		-	**91628**	**45185–185463**	***
8	Equine Clinic, Internal Medicine		Corridor	12	1		127	<LOD-834			0	**6423**		**2817–14560**		***	-		**69440**		**34620–139568**		***	12		-	**117243**	**63896–215227**	***
9	Ruminant Clinic		Lecture hall	12	10		5.5	<LOD—9.3			4	**123**		**52–287**		*	-		**3582**		**1791–7202**		***	12		-	**658**	**359–1213**	***
10	Clinic for Obstetrics		Lecture hall	12	7		8.6	<LOD-58.3			1	**232**		**101–530**		***	-		**3827**		**1910–7684**		***	10		-	**786**	**403–1526**	***
11			Locker room	10	4		16	<LOD-148			1	**649**		**260–1599**		***	-		**12664**		**5923–27211**		***	9		-	**4119**	**2044–8329**	***
12	Surgery lecture hall		Foyer	11	11		5.0	<LOD-<LOD			2	**108**		**45–260**		*	-		**2981**		**1441–6175**		***	11		-	**252**	**133–476**	***
13	Institute of Anatomy		Practice room	12	11		5.3	<LOD-9.3			9	13		3,9–35		ns	-		444		221–895		ns	12		-	56	31–103	ns
14			Microscopy room	12	11		5.5	<LOD-16.5			7	42		16–104		ns	-		999		497–2009		ns	12		-	40	22–74	ns
15			Lecture hall	10	11		5.0	<LOD-<LOD			3	90		34–228		ns	-		1083		504–2327		ns	10		-	58	30–113	ns
16			Locker room	11	8		5.7	<LOD-8.6			4	**158**		**64–383**		**	-		**2371**		**1139–4920**		***	9		-	59	29–118	ns
17	Institute of Physiology		Lecture hall	12	11		5.8	<LOD-27.3			4	80		33–188		ns	-		**1529**		**766–3078**		*	12		-	115	63–212	ns
18			Practice room	12	11		5.2	<LOD-7.9			7	22		8,1–56		ns	-		**2089**		**1037–4199**		***	12		-	**160**	**87–293**	***
19	Institute of Food Science		Practice room	11	10		5.2	<LOD-7.9			8	13		3,9–39		ns	-		**1553**		**752–3224**		*	12		-	49	26–94	ns
20	Institute of Pathology		Practice room	12	11		5.2	<LOD-7.9			7	52		202–12		ns	-		**6831**		**3397–13716**		***	12		-	**286**	**156–526**	***
21	Institute of Hygiene		Lecture hall	12	11		5.0	<LOD-<LOD			7	20		7,1–50		ns	-		687		344–1379		ns	12		-	32	17–58	ns
22			Practice room	12	12		5.0	<LOD-<LOD			9	14		4,5–39		ns	-		1013		505–2031		ns	12		-	43	23–78	ns
23	Deanery		Office	12	12		5.0	<LOD-<LOD			6	29		11–71		ns	-		**1814**		**900–3638**		**	12		-	67	37–124	ns
24	Learning Center		Computer room	10	9		5.3	<LOD-7.9			4	39		14–102		ns	-		**1855**		**868–3977**		**	10		-	66	34–129	ns
25	Faculty council		Office	12	11		5.2	<LOD-7.9			9	13		4,2–37		ns	-		**1617**		**802–3242**		*	12		-	67	36–123	ns
**CA**	Institute for Inorganic Chemistry		Laboratories	32	32		5.0	<LOD-<LOD			17	30		17–54			-		530		346–811			32		-	46	32–67	

CA: control area, N: number of measurements, ND: number of non-detectable samples, GM: geometric mean, PE: point estimate, CI: credible interval, BHT: Bayesian hypothesis test

***: pd>99.9% (certainly existing) **&** <1% in ROPE (significant)

**: pd>99.9% (certainly existing) **&** <2.5% in ROPE (probably significant) **OR** pd>99% (probably existing) & <1% in ROPE (significant)

*: pd>99% (probably existing) **&** <2.5% in ROPE (probably significant)

ns: not significant

In general, besides of mouse allergens all other determinants were found in almost all locations on the campus and in the control area, but in extremely variable concentrations. As expected, the highest levels of animal allergens (feline, canine, equine and bovine) were detected in buildings and rooms where the corresponding species were examined. Accordingly, Fel d 1 and Can f 1 concentrations were the highest in ultrasound, anesthesia, X-ray and seminar room within both Small Animal Clinics. However, at each location the Fel d 1 values were approximately 4-10-times lower than Can f 1 values. Equ c 1 levels were the highest in the rooms of the Horse Clinics and Clinic for Obstetrics. Interestingly, Equ c 1 levels were almost always higher than for other allergens at all other locations without presence of horses. The highest concentrations of Bos d 2 were found in the rooms of the Ruminant Clinic and the Clinic for Obstetrics. Locations where large animals were present (Horse Clinics and Clinic for Obstetrics) had the highest levels of mouse and DM allergens as well as β-(1,3)-glucan and endotoxin. In the absence of the respective animal species in buildings or rooms, allergen levels were drastically lower, but still elevated in some locations compared to the control area. In total, 20 of 25 sites examined on the campus were elevated for β-(1,3)-glucan, 17 for Equ c 1, 16 for Can f 1, 13 for DM allergens, ten for endotoxin, nine for Fel d 1 and Bos d 2 and three for Mus m 1 compared to the control area. The least contaminated areas included the rooms of the Institute of Hygiene, the Institute of Food Science and the Institute for Anatomy (with the exception of the locker room).

**Fig 1:**
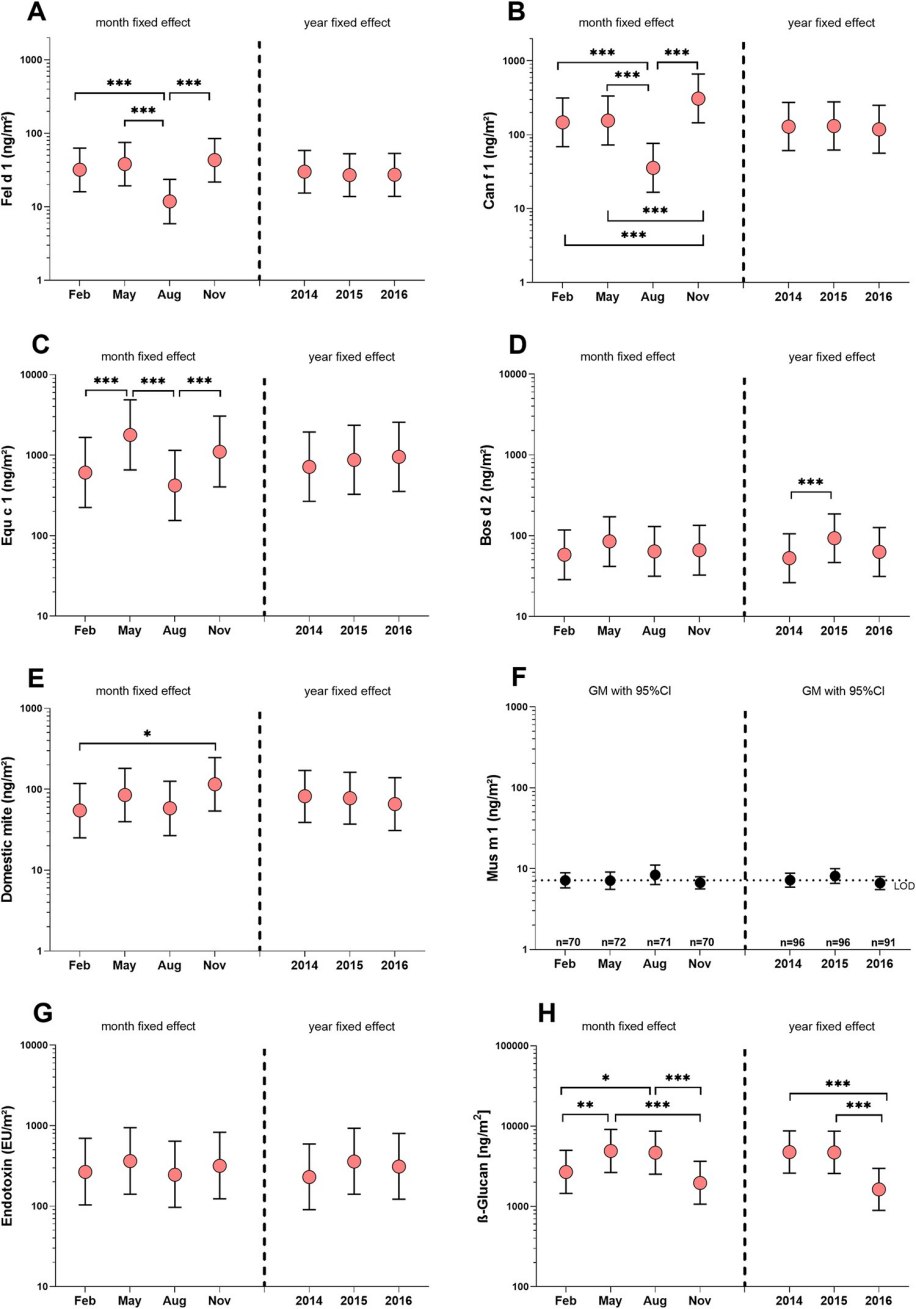
Seasonal and annual variations of concentrations of A) cat allergen, B) dog allergen, C) horse allergen, D) cattle allergen, E) domestic mite allergens, F) mouse allergen (only descriptive measures), G) β-(1,3)-glucan and H) endotoxin. Following parameters of the Bayesian models are shown: the point estimate—of the posteriors—as an index of centrality (red circle) and the 95% credible interval characterizing the dispersion of the posteriors (whiskers). The results of Bayesian hypothesis tests are marked with asterisks: ***: pd>99.9% (certainly existing) **&** <1% in ROPE (significant), **: pd>99.9% (certainly existing) **&** <2.5% in ROPE (probably significant) **OR** pd>99% (probably existing) & <1% in ROPE (significant), *: pd>99% (probably existing) **&** <2.5% in ROPE (probably significant), The exact pd and ROPE values are listed in S3 Table.

Fig 1 shows the seasonal and annual differences in the concentrations of all bio-contaminants. Influence of the season was observed for Fel d 1, Can f 1, Equ c 1 and β-(1,3)-glucan. For the corresponding allergens, the data showed a common significant seasonal effect with lowest values in August. Furthermore, Can f 1 was found to be elevated in November and Equ c 1 in May. For β-(1,3)-glucan, the levels in February and in November were significantly lower than in May and August. Almost the same seasonal effects were observed in the control area (S1 Fig). In general, variations between years were much less apparent than between seasons. A significant impact of the year was determined for β-(1,3)-glucan that was 3-times lower in 2016 than 2014 or 2015. Very small difference was observed for Bos d 2 between 2014 and 2015.

**Fig 2:**
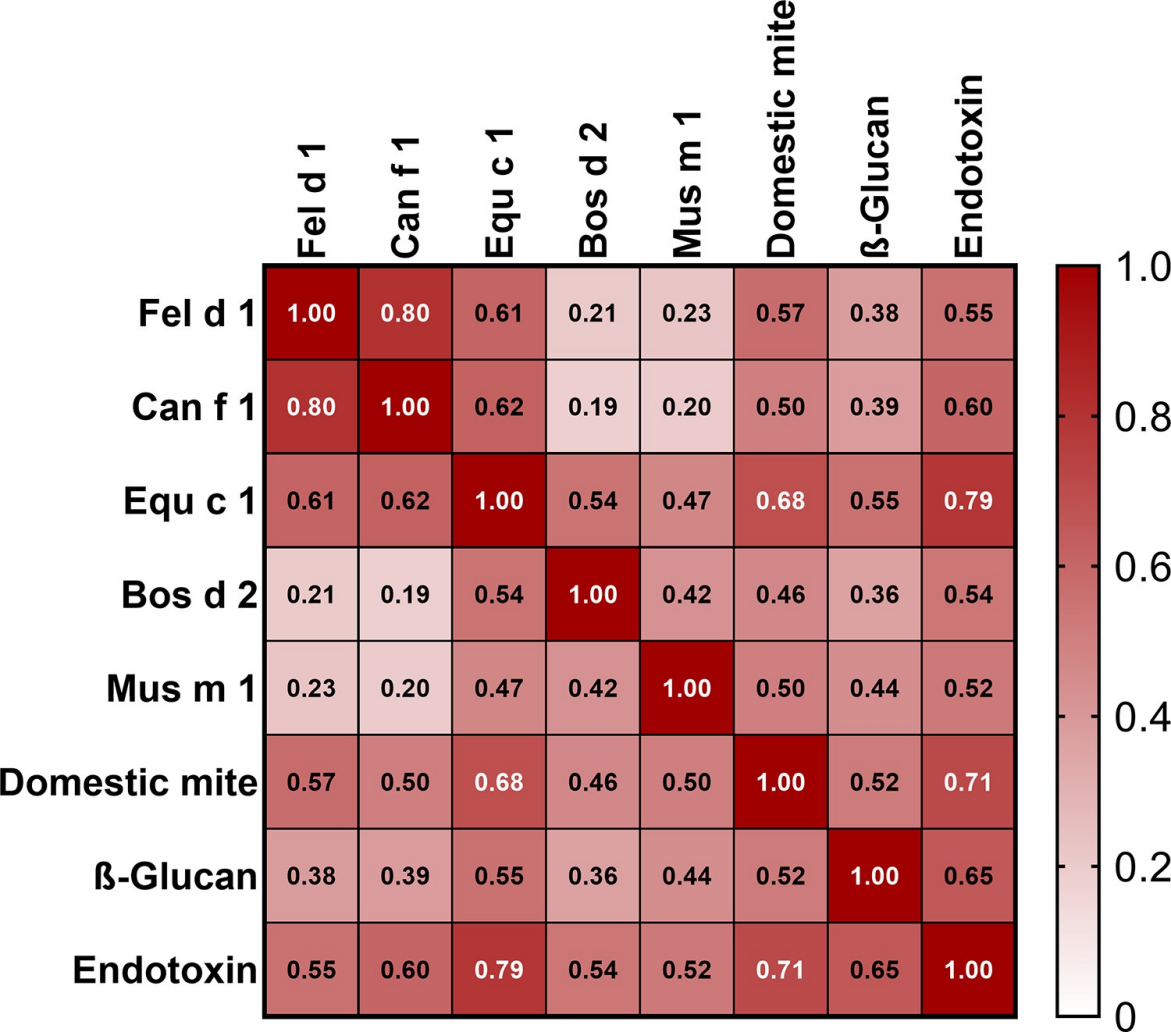
Heatmap of the Spearman correlation matrix of all bio-contaminant concentrations.

The correlation analysis revealed highly significant correlations of varying degrees between all analytes (Fig 2). The highest correlation was between cat and dog allergens (r = 0.8), followed by correlations between Equ c 1, mite allergens and endotoxin (r between 0.68 and 0.71). Bovine and mouse allergens correlated most poorly with all other determinants (mostly r < 0.5).

## Discussion

### Mammalian allergens

#### Exposure levels

With the exception of mouse allergens, all other mammalian allergens were found in all locations at the university campus as well as in the animal-free control area. The ubiquitous presence of animal allergens is not unexpected because based on their aerodynamic properties, mammalian allergens easily become airborne and can be simply spread from one environment to another. There is strong evidence that human clothing is the main carrier of allergens [36, 37]. In our study, this assumption can be supported by elevated animal allergen values found in the locker room of the Institute of Anatomy (location 16), an area that was otherwise not contaminated.

As expected, the concentrations of the respective allergen were the highest in buildings where the respective animal species were examined. It is well known, that the presence of animals is the strongest predictor of animal allergen levels. Homes with dogs or cats have usually 50-100-times higher Can f 1 or Fel d 1 levels than homes without these animals [2, 12, 38–40]. Levels of cattle or horse allergens in stables are even 500–5.000-fold higher than in farmers`dwellings or other buildings located near the stables [4, 6, 41]. In our study, concentrations occasionally differed in similar high orders of magnitude. For example, Can f 1 and Fel d 1 levels in anesthesia room (location 3) were 85-fold higher than in surgery lecture hall (location 12), and Equ c 1 levels in examination room for horses (location 7) were 700-fold higher than in surgery lecture hall.

Cats and dogs were examined or housed in the same buildings, so that Fel d 1 and Can f 1 values were the best correlated of all other determinants (r = 0.8). It was noticeable that at each location, regardless of the presence of the animals, Can f 1 values were usually 3-fold to 10-fold higher than the Fel d 1 values. There are several possible reasons for the much higher Can f 1 levels. Firstly, dogs were treated more frequently in the clinics compared to cats, although this could not be proven by official statistics (estimate of the employees). Secondly, unlike dogs, cats are brought to the clinic in boxes, so they cannot spread as many allergens in the environment as dogs. Thirdly, students and university staff often bring their own dogs to class or work, even though this is not actually allowed (communication from the faculty management). In addition, dogs might release more allergens than cats due to their higher average size. This theory is supported by the results of the German study in small animal practices, where Can f 1 levels were approximately twice as high as Fel d 1 values in every type of room, although similar percentages of cats (40%) and dogs (44%) were examined [2]. Albeit not always the same room types were sampled, the pet allergen levels (Fel d 1 and Can f 1) tended to be higher in the Small Animal Clinic in our study than in private small animal practices. This is probably due to the fact that in student education centers more people are working in a smaller space at the same time, resulting in higher air and dust turbulence. In private veterinary practices allergen levels decreased significantly with lower occupancy [2].

The horse allergen Equ c 1 was found most frequently of all allergens and also in the highest concentrations, even in the control area. This would again support the theory that the size of the animals has a strong influence on the amount of allergen released to the environment. However, this conclusion is not transferable to the cattle allergens. Although horses and cattle are similar in size and comparably abundant on the campus, the concentrations of bovine allergen Bos d 2 were much lower than Equ c 1 and were generally in the range of the control area (except the buildings with cattle). In contrast, Equ c 1 concentrations were mostly elevated compared to the control area. A wide distribution of Equ c 1 has also been shown in small animal veterinary practices, whereas Bos d 2 was rarely detected [2]. Hence, there are probably differences in the environmental dispersal of these two allergens. Much larger proportion of the general population has contact with horses compared to cattle, as horseback riding is a popular recreational sport. According to a 2016 study by the Allensbach Market and Advertising Media Analysis (AWA), 3.89 million people (4.7%) over the age of 14 in Germany describe themselves as riders [42]. The contact with cattle is mostly work-related and not as close as with horses. Regular grooming of horses also enhances the environmental spread of allergens. In addition, horses are one of the few mammalian species that can sweat [43]. By wetting the coat and subsequent drying, the allergens could be released into the air more easily. However, these properties of allergens still need to be determined.

The mouse allergen Mus m 1 was the rarest of all allergens and, when positive, usually in very low concentrations. This is probably due to the fact that mice are hardly ever kept as pets and are therefore very rarely examined in animal clinics. Detectable Mus m 1 levels were measured only in buildings where horses were examined. At these locations, the elevated Mus m 1 concentrations could be due to the transfer of allergens from the stables, where mice find sufficient food supply and favorable living conditions. However, Mus m 1 concentrations on EDC in mouse rooms of a laboratory animal facility were much higher (median of 100 ng/cloth corresponding to 5000 ng/m^2^) [44] than the highest levels measured in our study.

#### Seasonality

Seasonal effects were observed in our study for Fel d 1, Can f 1 and Equ c 1. The maximum difference between the lowest and highest seasonal values was 8-times for Can f 1 and 4-times for Fel d 1 and Equ c 1. Thus, the season was a much weaker predictor for animal allergen concentrations compared to the presence of animals.

Even if very rarely examined, seasonality was also found to have an influence on animal allergens in other studies reporting higher pet allergen levels in autumn and winter and lowest levels in summer [11, 12, 45, 46]. In contrast bovine and horse allergens were higher in summer than in winter [4, 6]. The authors offered several explanations for the seasonality, including fur shedding cycles or the fact that pets spend more time indoors during the colder months, or that more rain in autumn and frozen grounds in winter lead to lower concentrations of horse allergens. However, the factors that might control the seasonality of animal allergens remain unclear, and the differences between the seasons in these studies were in maximum about 2-fold. Some of the effects already described were also found in our study, e.g. highest Can f 1 levels in November or highest levels of Equ c 1 in May. However, the clearest effect with the lowest values in August was observed uniformly for Fel d 1, Can f 1 and Equ c 1. In our opinion, the lower occupancy and the lower utilization of the rooms are the most likely reasons for the significantly lower allergen levels in August, which is in the middle of the semester break at German universities. However, this effect was not observed for bovine allergens. As already mentioned, contact to cattle is not as close as to dog, cat and horses and is therefore less influenced by the presence and behavior of humans.

Compared to the seasonal variations, no significant changes in animal allergen levels were found between years (except of Bos d 2 with a negligible deviation). This shows that the conditions on the campus repeat more or less uniformly every year.

### Ubiquitous agents (domestic mite allergens, endotoxin and β-(1,3)-glucan)

#### Exposure levels

Similar to mouse allergens, the highest levels of DM allergens, endotoxin and β-(1,3)-glucan were found in the Equine Clinics, most likely through the transfer from the stables. The concentrations were about 100-times higher for DM allergens, about 400-times higher for β-(1,3)-glucan and 2000-times higher for endotoxin compared to the control area. Investigations of acarofauna in various European countries have shown high population densities of numerous mite species in diverse farm buildings [47–49]. Several studies conducted in different animal clinics have also shown that substantial bio-aerosol exposure (inhalable dust, endotoxin and β-(1,3)-glucan) occurs especially when handling farm animals and horses. The exposure levels were comparable to those observed in farming environments and much lower than during work with companion animals [3]. High exposure to these pro-inflammatory agents may be one possible reason for the increasing onset of self-reported respiratory symptoms without evidence of positive IgE among veterinary students handling farm animals [22].

Although considerably lower than in the Equine Clinic, the levels of DM allergens, endotoxin and β-(1,3)-glucan in examination rooms of the Small Animal Clinic were clearly elevated compared to the locations of different institutes, where the levels of these three components can be probably considered as a background exposure. It is known from studies in residential areas that the presence of pets (cats and/or dogs) leads to significantly increased endotoxin levels [50, 51], while no effects were found for DM allergen concentrations [11, 12]. This has not yet been studied for β-(1,3)-glucan. The levels of the three ubiquitous agents measured in the Small Animal Clinic were also higher than those determined in rooms occupied by animals in private veterinary practices for small animals (77 ng/m^2^ for DM, 150 EU/m^2^ for endotoxin and 2550 ng/m^2^ for β-(1,3)-glucan) [2]. The reason for this is probably similar to that for animal allergens: higher occupancy and activity in the rooms increases air and dust disturbance as well as the transfer of bio-contaminants from other sites.

#### Seasonality

Unlike the seasonality observed for most mammalian allergens, there was no common effect of the season on ubiquitous agents. Each component showed a different pattern. DM allergen concentrations were similar among years, but peaked in November, although the effect was weak. Varying with seasonal humidity, the highest dust mite populations occur during the humid summer months and the lowest numbers during the dry winter months [52]. The time lag between peak allergen and peak dust-mite populations is attributed to persistence of allergen in the environment. Most of the mites die during the winter, but dust containing allergens is stirred up by heating systems [45]. Endotoxin concentrations were more or less constant among seasons and years. This is in line with other investigations. Frankel et al. studied seasonal variations of diverse indoor and outdoor microbial components and their relation to temperature, relative humidity and air exchange rate and found significant seasonal differences for all measured exposures, excluding indoor endotoxin [53]. Similar results for airborne endotoxin were also described in other studies [54–56]. In contrast to endotoxin, the season has been determined to be an important factor for airborne β-(1,3)-glucan [57, 58] as well as for the much better studied airborne fungi in both indoors and outdoors [53, 59, 60]. Seasonal variations cause a regular pattern of variation in airborne fungi levels in many different climatic regions. Typically, concentrations are the lowest in winter and highest in summer. This is consistent with our results, which show higher concentrations in warm months (May and August) than in cold ones (February and November). There is also strong evidence that outdoor air dominates the composition of indoor air [53, 61]. It is also evident, that fungal growth is highly dependent on local environmental conditions and climatic factors such as temperature and humidity. This is probably the reason why β-(1,3)-glucan was the only component that showed significant variation between years.

## Conclusions

The multiple monitoring of allergen exposure over a longer period of time (three years in four seasons) showed considerable variations between and within sampled locations. The variations between collection sites are primarily due to the presence of the respective animals at the location. The season also affects the levels of various bio-contaminants, although the effects are much smaller compared to the presence of animals. Furthermore, the measurements showed that exposure ranges are repeated year by year. In addition, the passive transfer from stable environment, especially regarding horses, is a relevant factor for spreads of allergens, endotoxin and glucan.

For the assessment of the hazard potential of allergens, endotoxins and β-(1–3)-glucans, neither exposure limits nor dose-response relationships are currently available. Furthermore, estimation of “general” risk values for these agents may not be possible due to strong dependence on the individual susceptibility. Allergen levels associated with increased risk of disease are certainly different for healthy, sensitized or allergic persons. However, knowledge of the level of exposure and the factors influencing exposure can be helpful in assessing risk for specific workplaces. The type and extent of exposure are decisive for the degree of risk. If representative values for background exposure and maximum exposure are available, then a classification of concentrations of bio-contaminants into “elevated”, “high”, or “very high” is possible. Our study based on a relatively large sample size, standardized dust collection and assessment of relevant allergens together with other bio-components in parallel can contribute to a better classification of the concentrations of these agents.

## Supporting information

S1 Appendix (DOCX)

S1 Table Results of Bayesian hypothesis tests—comparison of animal allergen, endotoxin and β-(1,3)-glucan levels at different locations of the veterinary medicine campus with a control area. CA: control area, pd: probability of direction, Rope: region of practical equivalence.
(DOCX)

S2 Table Animal allergen, endotoxin and β-(1,3)-glucan levels at different locations of the veterinary medicine campus (descriptive statistics). CA: control area, N: number of measurements, ND: number of non-detectable samples, GM: geometric mean, GSD: geometric standard deviation.
(DOCX)

S3 Table Results of Bayesian hypothesis tests—comparison of seasonal and annual variations of animal allergen, endotoxin and β-(1,3)-glucan levels. pd: probability of direction, Rope: region of practical equivalence.
(DOCX)

S1 Fig Seasonal variations of allergen, endotoxin and β-(1,3)-glucan concentrations (GM with 95% CI) in the animals-free control area. (TIF)

## References

[1] Samadi S, Heederik DJJ, Krop EJM, Jamshidifard A-R, Willemse T, Wouters IM. Allergen and endotoxin exposure in a companion animal hospital. Occup Environ Med. 2010; 67:486–92. doi: 10.1136/oem.2009.051342 . 20519747

[2] Zahradnik E, Sander I, Kleinmüller O, Lotz A, Liebers V, Janssen-Weets B, et al. Animal Allergens, Endotoxin, and β-(1,3)-Glucan in Small Animal Practices: Exposure Levels at Work and in Homes of Veterinary Staff. Annals of Work Exposures and Health. 2021. Epub 2021/08/07. doi: 10.1093/annweh/wxab053 . 34363388 PMC8751790

[3] Samadi S, Wouters IM, Heederik DJJ. A review of bio-aerosol exposures and associated health effects in veterinary practice. Ann Agric Environ Med. 2013; 20:206–21. 23772565

[4] Schlünssen V, Basinas I, Zahradnik E, Elholm G, Wouters IM, Kromhout H, et al. Exposure levels, determinants and IgE mediated sensitization to bovine allergens among Danish farmers and non-farmers. Int J Hyg Environ Health. 2015; 218:265–72. doi: 10.1016/j.ijheh.2014.12.002 . 25534699

[5] Zahradnik E, Sander I, Kendzia B, Fleischer C, Brüning T, Raulf-Heimsoth M. Passive airborne dust sampling to assess mite antigen exposure in farming environments. J Environ Monit. 2011; 13:2638–44. Epub 2011/08/15. doi: 10.1039/c1em10430f . 21842065

[6] Elfman L, Brannstrom J, Smedje G. Detection of horse allergen around a stable. Int Arch Allergy Immunol. 2008; 145:269–76. Epub 2007/11/15. doi: 10.1159/000110885 . 18025788

[7] Stave GM. Occupational Animal Allergy. Curr Allergy Asthma Rep. 2018; 18:11. Epub 2018/02/16. doi: 10.1007/s11882-018-0755-0 . 29453631

[8] Nilsson OB, van Hage M, Grönlund H. Mammalian-derived respiratory allergens—implications for diagnosis and therapy of individuals allergic to furry animals. Methods. 2014; 66:86–95. Epub 2013/09/14. doi: 10.1016/j.ymeth.2013.09.002 . 24041755

[9] Calderón MA, Linneberg A, Kleine-Tebbe J, Blay F de, Hernandez Fernandez de Rojas D, Virchow JC, et al. Respiratory allergy caused by house dust mites: What do we really know. J Allergy Clin Immunol. 2015; 136:38–48. Epub 2014/11/22. doi: 10.1016/j.jaci.2014.10.012 . 25457152

[10] Sander I, Zahradnik E, Kraus G, Mayer S, Neumann H-D, Fleischer C, et al. Domestic mite antigens in floor and airborne dust at workplaces in comparison to living areas: a new immunoassay to assess personal airborne allergen exposure. PLoS ONE. 2012; 7:e52981. doi: 10.1371/journal.pone.0052981 . 23285240 PMC3528730

[11] Sander I, Lotz A, Neumann HD, Czibor C, Flagge A, Zahradnik E, et al. Indoor allergen levels in settled airborne dust are higher in day-care centers than at home. Allergy. 2018; 73:1263–75. doi: 10.1111/all.13371 . 29193190

[12] Krop EJM, Jacobs JH, Sander I, Raulf-Heimsoth M, Heederik DJJ. Allergens and β-Glucans in Dutch Homes and Schools: Characterizing Airborne Levels. PLoS ONE. 2014; 9:e88871. doi: 10.1371/journal.pone.0088871 24551183 PMC3925184

[13] Sander I, Lotz A, Liebers V, Zahradnik E, Sauke-Gensow U, Petersen J, et al. Comparing the concentration levels of allergens and endotoxins in employees’ homes and offices. Int Arch Occup Environ Health. 2022; 95:573–88. Epub 2021/11/05. doi: 10.1007/s00420-021-01794-9 . 34738178 PMC8938351

[14] Eduard W, Heederik D, Duchaine C, Green BJ. Bioaerosol exposure assessment in the workplace: the past, present and recent advances. J Environ Monit. 2012; 14:334–9. Epub 2012/01/23. doi: 10.1039/c2em10717a . 22267210 PMC4687010

[15] Bonini S, Buonacucina A, Selis L, Peli A, Mutti A, Corradi M. Occupational Hazards in Veterinarians: An Updating. J Veterinar Sci Technol. 2016; 07. doi: 10.4172/2157-7579.1000317

[16] Elbers AR, Blaauw PJ, Vries M de, van Gulick PJ, Smithuis OL, Gerrits RP, et al. Veterinary practice and occupational health. An epidemiological study of several professional groups of Dutch veterinarians. I. General physical examination and prevalence of allergy, lung function disorders, and bronchial hyperreactivity. Vet Q. 1996; 18:127–31. doi: 10.1080/01652176.1996.9694711 . 8972059

[17] Jeyaretnam J, Jones H, Phillips M. Disease and injury among veterinarians. Aust Vet J. 2000; 78:625–9. doi: 10.1111/j.1751-0813.2000.tb11939.x . 11022291

[18] Susitaival P, Kirk JH, Schenker MB. Atopic symptoms among California veterinarians. Am J Ind Med. 2003; 44:166–71. doi: 10.1002/ajim.10253 . 12874849

[19] Nienhaus A, Skudlik C, Seidler A. Work-related accidents and occupational diseases in veterinarians and their staff. Int Arch Occup Environ Health. 2005; 78:230–8. doi: 10.1007/s00420-004-0583-5 . 15776262

[20] Epp T, Waldner C. Occupational health hazards in veterinary medicine: zoonoses and other biological hazards. Can Vet J. 2012; 53:144–50. 22851775 PMC3258827

[21] Schelkle M, Braun J, Jörres R, Schierl R, Dressel H. Respiratory allergies among veterinarians: two cross-sectional surveys from 2006 to 2012. Int Arch Occup Environ Health. 2017; 90:639–43. Epub 2017/05/06. doi: 10.1007/s00420-017-1226-y . 28478545

[22] Samadi S, Spithoven J, Jamshidifard A-R, Berends BR, Lipman L, Heederik DJJ, et al. Allergy among veterinary medicine students in The Netherlands. Occup Environ Med. 2012; 69:48–55. Epub 2011/05/31. doi: 10.1136/oem.2010.064089 . 21632519

[23] Raulf M, Buters J, Chapman M, Cecchi L, Blay F de, Doekes G, et al. Monitoring of occupational and environmental aeroallergens—EAACI Position Paper. Concerted action of the EAACI IG Occupational Allergy and Aerobiology & Air Pollution. Allergy. 2014; 69:1280–99. Epub 2014/08/04. doi: 10.1111/all.12456 . 24894737

[24] Sander I, Lotz A, Zahradnik E, Raulf M. Allergen Quantification by Use of Electrostatic Dust Collectors (EDCs): Influence of Deployment Time, Extraction Buffer, and Storage Conditions on the Results. Ann Occup Hyg. 2016; 60:845–59. doi: 10.1093/annhyg/mew027 . 27229526

[25] Zahradnik E, Sander I, Brüning T, Raulf M. Allergen Levels in the Hair of Different Cattle Breeds. Int Arch Allergy Immunol. 2015; 167:9–15. doi: 10.1159/000431227 . 26087837

[26] Zahradnik E, Janssen-Weets B, Sander I, Kendzia B, Mitlehner W, May C, et al. Lower allergen levels in hypoallergenic Curly Horses? A comparison among breeds by measurements of horse allergens in hair and air samples. PLoS ONE. 2018; 13:e0207871. doi: 10.1371/journal.pone.0207871 . 30540798 PMC6291085

[27] Sander I, Fleischer C, Borowitzki G, Brüning T, Raulf-Heimsoth M. Development of a two-site enzyme immunoassay based on monoclonal antibodies to measure airborne exposure to (1–3)-beta-D-glucan. J Immunol Methods. 2008; 337:55–62. doi: 10.1016/j.jim.2008.05.010 . 18589436

[28] Liebers V, van Kampen V, Bünger J, Düser M, Stubel H, Brüning T, et al. Assessment of airborne exposure to endotoxin and pyrogenic active dust using electrostatic dustfall collectors (EDCs). J Toxicol Environ Health Part A. 2012; 75:501–7. doi: 10.1080/15287394.2012.674919 . 22686309

[29] Lunn DJ, Thomas A, Best N, Spiegelhalter D. WinBUGS—A Bayesian modelling framework: Concepts, structure, and extensibility. Statistics and Computing. 2000; 10:325–37. doi: 10.1023/A:1008929526011.

[30] R Core Team. R: A Language and Environment for Statistical Computing. Vienna, Austria: R Foundation for Statistical Computing: 2020. Available from: http://www.R-project.org.

[31] Makowski D, Ben-Shachar M, Lüdecke D. bayestestR: Describing Effects and their Uncertainty, Existence and Significance within the Bayesian Framework. JOSS. 2019; 4:1541. doi: 10.21105/joss.01541 PMC6914840 31920819

[32] Magnusson Arni, Stewart Ian J. plotMCMC: MCMC diagnostic plots. 2005. Available from: https://CRAN.R-project.org/package=plotMCMC.

[33] Sturtz S, Ligges U, Gelman A. R2WinBUGS: A Package for Running WinBUGS from R. J Stat Soft. 2005; 12. doi: 10.18637/jss.v012.i03

[34] Makowski D, Ben-Shachar MS, Chen SHA, Lüdecke D. Indices of Effect Existence and Significance in the Bayesian Framework. Front Psychol. 2019; 10:2767. Epub 2019/12/10. doi: 10.3389/fpsyg.2019.02767 . 31920819 PMC6914840

[35] Reporting Guidelines: How to describe and report the parameters of a model [updated 8 Feb 2023]. Available from: https://easystats.github.io/bayestestR/articles/guidelines.html.

[36] De Lucca SD O’meara TJ, Tovey ER. Exposure to mite and cat allergens on a range of clothing items at home and the transfer of cat allergen in the workplace. Journal of Allergy and Clinical Immunology. 2000; 106:874–9. doi: 10.1067/mai.2000.110804 . 11080709

[37] Egmar AC, Almqvist C, Emenius G, Lilja G, Wickman M. Deposition of cat (Fel d 1), dog (Can f 1), and horse allergen over time in public environments—a model of dispersion. Allergy. 1998; 53:957–61. doi: 10.1111/j.1398-9995.1998.tb03796.x . 9821475

[38] Sander I, Neumann H-D, Lotz A, Czibor C, Zahradnik E, Flagge A, et al. Allergen quantification in surface dust samples from German day care centers. J Toxicol Environ Health Part A. 2016; 79:1094–105. doi: 10.1080/15287394.2016.1219597 . 27924716

[39] Heinrich J, Bedada GB, Zock J-P, Chinn S, Norbäck D, Olivieri M, et al. Cat allergen level: its determinants and relationship to specific IgE to cat across European centers. J Allergy Clin Immunol. 2006; 118:674–81. doi: 10.1016/j.jaci.2006.06.012 . 16950287

[40] Arbes SJ, Cohn RD, Yin M, Muilenberg ML, Friedman W, Zeldin DC. Dog allergen (Can f 1) and cat allergen (Fel d 1) in US homes: Results from the National Survey of Lead and Allergens in Housing. Journal of Allergy and Clinical Immunology. 2004; 114:111–7. doi: 10.1016/j.jaci.2004.04.036 15241352

[41] Zahradnik E, Sander I, Bruckmaier L, Flagge A, Fleischer C, Schierl R, et al. Development of a sandwich ELISA to measure exposure to occupational cow hair allergens. Int Arch Allergy Immunol. 2011; 155:225–33. doi: 10.1159/000319839 . 21282961

[42] Press release of the German Equestrian Federation. [Figures, data, facts from equestrian sport and horse breeding 2021] [updated 22 Nov 2022]. Available from: https://www.pferd-aktuell.de/shop/downloadable/download/sample/sample_id/224/.

[43] McDonald RE, Fleming RI, Beeley JG, Bovell DL, Lu JR, Zhao X, et al. Latherin: a surfactant protein of horse sweat and saliva. PLoS ONE. 2009; 4:e5726. Epub 2009/05/29. doi: 10.1371/journal.pone.0005726 . 19478940 PMC2684629

[44] Feistenauer S, Sander I, Schmidt J, Zahradnik E, Raulf M, Brielmeier M. Influence of 5 different caging types and the use of cage-changing stations on mouse allergen exposure. J Am Assoc Lab Anim Sci. 2014; 53:356–63. 25199090 PMC4113234

[45] Chew GL, Higgins KM, Gold DR, Muilenberg ML, Burge HA. Monthly measurements of indoor allergens and the influence of housing type in a northeastern US city. Allergy. 1999; 54:1058–66. doi: 10.1034/j.1398-9995.1999.00003.x . 10536884

[46] Heinrich J, Hölscher B, Douwes J, Richter K, Koch A, Bischof W, et al. Reproducibility of allergen, endotoxin and fungi measurements in the indoor environment. J Expo Anal Environ Epidemiol. 2003; 13:152–60. doi: 10.1038/sj.jea.7500267 . 12679795

[47] Solarz K, Pająk C. Risk of exposure of a selected rural population in South Poland to allergenic mites. Part II: acarofauna of farm buildings. Exp Appl Acarol. 2019; 77:387–99. Epub 2019/03/05. doi: 10.1007/s10493-019-00355-7 . 30835019

[48] Franz JT, Masuch G, Müsken H, Bergmann KC. Mite fauna of German farms. Allergy. 1997; 52:1233–7. doi: 10.1111/j.1398-9995.1997.tb02529.x . 9450144

[49] Terho EO, Leskinen L, Husman K, Kärenlampi L. Occurrence of storage mites in Finnish farming environments. Allergy. 1982; 37:15–9. doi: 10.1111/j.1398-9995.1982.tb04112.x . 7137517

[50] Mendy A, Wilkerson J, Salo PM, Cohn RD, Zeldin DC, Thorne PS. Exposure and Sensitization to Pets Modify Endotoxin Association with Asthma and Wheeze. The Journal of Allergy and Clinical Immunology: In Practice. 2018; 6:2006–2013.e4. doi: 10.1016/j.jaip.2018.04.009 29684578 PMC6524530

[51] Heinrich J, Gehring U, Douwes J, Koch A, Fahlbusch B, Bischof W, et al. Pets and vermin are associated with high endotoxin levels in house dust. Clin Exp Allergy. 2001; 31:1839–45. doi: 10.1046/j.1365-2222.2001.01220.x . 11737034

[52] Miller JD. The Role of Dust Mites in Allergy. Clin Rev Allergy Immunol. 2019; 57:312–29. doi: 10.1007/s12016-018-8693-0 . 29936683

[53] Frankel M, Bekö G, Timm M, Gustavsen S, Hansen EW, Madsen AM. Seasonal variations of indoor microbial exposures and their relation to temperature, relative humidity, and air exchange rate. Appl Environ Microbiol. 2012; 78:8289–97. Epub 2012/09/21. doi: 10.1128/AEM.02069-12 . 23001651 PMC3497365

[54] Park JH, Spiegelman DL, Gold DR, Burge HA, Milton DK. Predictors of airborne endotoxin in the home. Environ Health Perspect. 2001; 109:859–64. doi: 10.1289/ehp.01109859 . 11564624 PMC1240416

[55] Madsen AM, Matthiesen CB, Frederiksen MW, Frederiksen M, Frankel M, Spilak M, et al. Sampling, extraction and measurement of bacteria, endotoxin, fungi and inflammatory potential of settling indoor dust. J Environ Monit. 2012; 14:3230–9. Epub 2012/11/15. doi: 10.1039/c2em30699a . 23152160

[56] Kilburg-Basnyat B, Peters TM, Perry SS, Thorne PS. Electrostatic dust collectors compared to inhalable samplers for measuring endotoxin concentrations in farm homes. Indoor Air. 2016; 26:724–33. Epub 2015/09/21. doi: 10.1111/ina.12243 . 26296624 PMC4850132

[57] Madsen AM, Frederiksen MW, Allermann L, Peitersen JH. (1 → 3)-β-d-glucan in different background environments and seasons. Aerobiologia. 2011; 27:173–9. doi: 10.1007/s10453-010-9178-7

[58] Thorn J. Seasonal variations in exposure to microbial cell wall components among household waste collectors. Ann Occup Hyg. 2001; 45:153–6. 11182429

[59] Shelton BG, Kirkland KH, Flanders WD, Morris GK. Profiles of airborne fungi in buildings and outdoor environments in the United States. Appl Environ Microbiol. 2002; 68:1743–53. doi: 10.1128/AEM.68.4.1743-1753.2002 . 11916692 PMC123871

[60] Nevalainen A, Täubel M, Hyvärinen A. Indoor fungi: companions and contaminants. Indoor Air. 2015; 25:125–56. Epub 2015/01/20. doi: 10.1111/ina.12182 . 25601374

[61] Adams RI, Miletto M, Taylor JW, Bruns TD. Dispersal in microbes: fungi in indoor air are dominated by outdoor air and show dispersal limitation at short distances. ISME J. 2013; 7:1262–73. Epub 2013/02/21. doi: 10.1038/ismej.2013.28 . 23426013 PMC3695294

