# Supplementary material for: Exposure levels of animal allergens, endotoxin, and β-(1,3)-glucan on a university campus of veterinary medicine
Source: PLoS One. 2023 Jul 13;18(7):e0288522. doi: 10.1371/journal.pone.0288522 (PMC10343150; doi:10.1371/journal.pone.0288522)
Supplement: S1 Appendix — (DOCX) [file pone.0288522.s001.docx]

**Statistical analysis**

1**) Differences between each location and the control area**

In all models, the Markov chain Monte Carlo (MCMC) sampling was conducted with three chains, each with 550.000 iterations, a burn-in of 50.000 iterations and a thinning of ten. MCMC chains were checked for convergence and resolution by visual inspection, demanding R-hat below 1.01, and demanding the effective number of draws (n.eff) above 10.000.

The simplified coding of the models in OpenBUGS including censoring is:

*model{
 for (1 in 1:315){
 y[i] ~ dnorm(mu[i], tau)C( ,LODcens[i])
 mu[i] <- beta0 + beta.season1 * season1[i] + beta.season 2 * season2[i] + beta.season 3 * season3[i] + beta.location1 * location1[i] + … + beta.location25 * location[i]
 }
 beta0 ~ dnorm(0, 0.0001)
 beta.season1 ~ dnorm(0, 0.0001) … beta.season3 ~ dnorm(0, 0.0001)
 beta.location1 ~ dnorm(0, 0.0001) … beta.location25 ~ dnorm(0, 0.0001)
 tau ~ dgamma(0.01, 0.01)
# generated parameters of interest
 y.location0.adjseason <- beta0 + 0.25 * beta.season1 + 0.25 * beta.season2 + 0.25 * beta.season3
 y.location1.adjseason <- beta0 + 0.25 * beta.season1 + 0.25 * beta.season2 + 0.25 * beta.season3 + beta.location1 …
 y.location25.adjseason <- beta0 + 0.25 * beta.season1 + 0.25 * beta.season2 + 0.25 * beta.season3 + beta.location25}*

**2) Differences between seasons and years**

In all models, the MCMC sampling was conducted with three chains, each with 75.000 iterations, a burn-in of 15.000 iterations and a thinning of three. Convergence of the chains were checked by visual inspection and demanding R-hat below 1.01, and the resolution was checked by demanding the effective number of draws (n.eff) above 10.000.
The simplified coding of the models in OpenBUGS including censoring is:
*model{
 for (1 in 1:283){
 y[i] ~ dnorm(mu[i], tau.e)C( ,LODcens[i])
 mu[i] <- beta[location[i]] + beta.season1 * season1[i] + beta.season 2 * season2[i] + beta.season 3 * season3[i] + beta.year1 * year1[i] + beta.year2 * year2[i]
 }
 for (j in 1:25) {beta[j] ~ dnorm(mu, tau.s)}
 beta.season1 ~ dnorm(0, 0.0001) … beta.season3 ~ dnorm(0, 0.0001)
 beta.year1 ~ dnorm(0, 0.0001)
 beta.year2 ~ dnorm(0, 0.0001)
 mu ~ dnorm(0, 0.0001)
 tau.s ~ dgamma(0.01, 0.01)
 tau.e ~ dgamma(0.01, 0.01)
# generated parameters of interest
 y.season0.adj <- mu + 1/3 * beta.year1 + 1/3 * beta.year2
 y.season1.adj <- mu + beta.season1 + 1/3 * beta.year1 + 1/3 * beta.year2
 y.season2.adj <- mu + beta.season2 + 1/3 * beta.year1 + 1/3 * beta.year2
 y.season3.adj <- mu + beta.season3 + 1/3 * beta.year1 + 1/3 * beta.year2
 y.year0.adj <- mu + 0.25 * beta.season1 + 0.25 * beta.season2 + 0.25 * beta.season3
 y.year1.adj <- mu + beta.year1 + 0.25 * beta.season1 + 0.25 * beta.season2 + 0.25 * beta.season3
 y.year2.adj <- mu + beta.year2 + 0.25 * beta.season1 + 0.25 * beta.season2 + 0.25 * beta.season3}*
