# Supplementary material for: Exposure levels of animal allergens, endotoxin, and β-(1,3)-glucan on a university campus of veterinary medicine
Source: PLoS One. 2023 Jul 13;18(7):e0288522. doi: 10.1371/journal.pone.0288522 (PMC10343150; doi:10.1371/journal.pone.0288522)
Supplement: S1 Table — CA: control area, pd: probability of direction, Rope: region of practical equivalence. (DOCX) [file pone.0288522.s002.docx]

**S1 Table.** **Results of Bayesian hypothesis tests - comparison of animal allergen, endotoxin and β-(1,3)-glucan levels at different locations of the veterinary medicine campus with a control area**

| **Location** | | | **Fel d 1 (ng/m²)** | | **Can f 1 (ng/m²)** | | **Equ c 1 (ng/m²)** | | **Bos d 2 (ng/m²)** | |
| --- | --- | --- | --- | --- | --- | --- | --- | --- | --- | --- |
| **No** | **Building** | **Room** | **pd** | **ROPE** | **pd** | **ROPE** | **pd** | **ROPE** | **pd** | **ROPE** |
| 1 | Small Animal Clinic, | Seminar room | >0.9999 | <0.0001 | >0.9999 | <0.0001 | >0.9999 | <0.0001 | 0.8524 | 0.2204 |
| 2 | Small Animal Clinic, Surgery | Ultrasound room | >0.9999 | <0.0001 | >0.9999 | <0.0001 | >0.9999 | <0.0001 | 0.9858 | 0.0410 |
| 3 |  | Anesthesia room | >0.9999 | <0.0001 | >0.9999 | <0.0001 | >0.9999 | <0.0001 | 0.9931 | 0.0219 |
| 4 |  | X-ray room | >0.9999 | <0.0001 | >0.9999 | <0.0001 | >0.9999 | <0.0001 | >0.9999 | <0.0001 |
| 5 |  | Inpatient ward | 0.9928 | 0.0183 | >0.9999 | <0.0001 | 0.5502 | 0.3571 | 0.9200 | 0.1206 |
| 6 |  | Exhibit collection | 0.9878 | 0.0367 | >0.9999 | <0.0001 | >0.9999 | <0.0001 | 0.7700 | 0.2928 |
| 7 | Equine Clinic, Surgery | Examination room | 0.9893 | 0.0275 | >0.9999 | 0.0003 | >0.9999 | <0.0001 | >0.9999 | <0.0001 |
| 8 | Equine Clinic, Internal Medicine | Corridor | 0.9977 | 0.0088 | >0.9999 | <0.0001 | >0.9999 | <0.0001 | >0.9999 | 0.0001 |
| 9 | Ruminant Clinic | Lecture hall | 0.7832 | 0.2626 | 0.9881 | 0.0459 | >0.9999 | <0.0001 | >0.9999 | <0.0001 |
| 10 | Clinic for Obstetrics | Lecture hall | 0.8592 | 0.2060 | >0.9999 | <0.0001 | >0.9999 | <0.0001 | >0.9999 | <0.0001 |
| 11 |  | Locker room | >0.9999 | <0.0001 | >0.9999 | <0.0001 | >0.9999 | <0.0001 | >0.9999 | <0.0001 |
| 12 | Surgery lecture hall | Foyer | 0.9984 | 0.0055 | >0.9999 | <0.0001 | >0.9999 | <0.0001 | 0.9934 | 0.0205 |
| 13 | Institute of Anatomy | Practice room | 0.9153 | 0.1407 | 0.8534 | 0.2628 | 0.5244 | 0.4156 | 0.6858 | 0.3271 |
| 14 |  | Microscopy room | 0.5168 | 0.3456 | 0.7988 | 0.3207 | 0.9160 | 0.1737 | 0.5972 | 0.3588 |
| 15 |  | Lecture hall | 0.9692 | 0.0628 | 0.9818 | 0.0574 | 0.9974 | 0.0113 | 0.5065 | 0.3422 |
| 16 |  | Locker room | >0.9999 | <0.0001 | >0.9999 | 0.0001 | >0.9999 | 0.0001 | 0.9726 | 0.0656 |
| 17 | Institute of Physiology | Lecture hall | 0.7814 | 0.2622 | 0.9999 | 0.0006 | 0.9940 | 0.0228 | 0.8568 | 0.2199 |
| 18 |  | Practice room | 0.6062 | 0.3427 | 0.9996 | 0.0027 | 0.9932 | 0.0259 | 0.9994 | 0.0024 |
| 19 | Institute of Food Science | Practice room | 0.6653 | 0.3109 | 0.6836 | 0.3782 | 0.7626 | 0.3151 | 0.9655 | 0.0781 |
| 20 | Institute of Pathology | Practice room | 0.9472 | 0.0963 | 0.9765 | 0.0718 | 0.7305 | 0.3495 | 0.9868 | 0.0397 |
| 21 | Institute of Hygiene | Lecture hall | 0.6904 | 0.3048 | 0.5691 | 0.4420 | 0.6328 | 0.3868 | 0.8709 | 0.2054 |
| 22 |  | Practice room | 0.8270 | 0.2356 | 0.8505 | 0.2721 | 0.9630 | 0.0965 | 0.7963 | 0.2696 |
| 23 | Deanery | Office | 0.9762 | 0.0522 | >0.9999 | <0.0001 | 0.9639 | 0.0965 | 0.5325 | 0.3657 |
| 24 | Learning Center | Computer room | 0.9698 | 0.0648 | 0.9563 | 0.1122 | 0.9998 | 0.0011 | 0.9646 | 0.0783 |
| 25 | Faculty council | Office | 0.7143 | 0.2952 | 0.9963 | 0.0182 | 0.9961 | 0.0163 | 0.8493 | 0.2309 |
| CA | Institute for Inorganic Chemistry | Laboratories | - | - | - | - | - | - | - | - |

CA: control area, pd: probability of direction, Rope: region of practical equivalence

**S1 Table (continued).** **Results of Bayesian hypothesis tests - comparison of animal allergen, endotoxin and β-(1,3)-glucan levels at different locations of the veterinary medicine campus with a control area**

| **Location** | | | **Domestic mite (ng/m^2^)** | | **β-(1,3)-glucan (ng/m²)** | | **Endotoxin (EU/m^2^)** | |
| --- | --- | --- | --- | --- | --- | --- | --- | --- |
| **No** | **Building** | **Room** | **pd** | **ROPE** | **pd** | **ROPE** | **pd** | **ROPE** |
| 1 | Small Animal Clinic, | Seminar room | 0.9534 | 0.0827 | >0.9999 | 0.0001 | >0.9999 | <0.0001 |
| 2 | Small Animal Clinic, Surgery | Ultrasound room | 0.9989 | 0.0039 | >0.9999 | <0.0001 | >0.9999 | <0.0001 |
| 3 |  | Anesthesia room | 0.9999 | 0.0004 | >0.9999 | <0.0001 | >0.9999 | <0.0001 |
| 4 |  | X-ray room | 0.9983 | 0.0058 | >0.9999 | <0.0001 | >0.9999 | <0.0001 |
| 5 |  | Inpatient ward | 0.9886 | 0.0173 | 0.9953 | 0.0120 | 0.7180 | 0.3612 |
| 6 |  | Exhibit collection | 0.7864 | 0.2167 | 0.9999 | 0.0007 | 0.9999 | 0.0009 |
| 7 | Equine Clinic, Surgery | Examination room | >0.9999 | <0.0001 | >0.9999 | <0.0001 | >0.9999 | <0.0001 |
| 8 | Equine Clinic, Internal Medicine | Corridor | >0.9999 | <0.0001 | >0.9999 | <0.0001 | >0.9999 | <0.0001 |
| 9 | Ruminant Clinic | Lecture hall | 0.9961 | 0.0109 | >0.9999 | <0.0001 | >0.9999 | <0.0001 |
| 10 | Clinic for Obstetrics | Lecture hall | >0.9999 | 0.0002 | >0.9999 | <0.0001 | >0.9999 | <0.0001 |
| 11 |  | Locker room | >0.9999 | <0.0001 | >0.9999 | <0.0001 | >0.9999 | <0.0001 |
| 12 | Surgery lecture hall | Foyer | 0.9913 | 0.0206 | >0.9999 | 0.0002 | >0.9999 | <0.0001 |
| 13 | Institute of Anatomy | Practice room | 0.9273 | 0.1004 | 0.6649 | 0.3166 | 0.7024 | 0.4273 |
| 14 |  | Microscopy room | 0.7162 | 0.2549 | 0.9356 | 0.1180 | 0.6484 | 0.4510 |
| 15 |  | Lecture hall | 0.9726 | 0.0501 | 0.9452 | 0.0967 | 0.7120 | 0.3943 |
| 16 |  | Locker room | 0.9987 | 0.0039 | 0.9997 | 0.0012 | 0.7183 | 0.3757 |
| 17 | Institute of Physiology | Lecture hall | 0.9662 | 0.0628 | 0.9945 | 0.0170 | 0.9937 | 0.0312 |
| 18 |  | Practice room | 0.7139 | 0.2530 | 0.9995 | 0.0022 | 0.9996 | 0.0033 |
| 19 | Institute of Food Science | Practice room | 0.9063 | 0.1155 | 0.9938 | 0.0178 | 0.5661 | 0.4632 |
| 20 | Institute of Pathology | Practice room | 0.8335 | 0.1932 | >0.9999 | <0.0001 | >0.9999 | <0.0001 |
| 21 | Institute of Hygiene | Lecture hall | 0.7833 | 0.2204 | 0.7339 | 0.2905 | 0.8558 | 0.2948 |
| 22 |  | Practice room | 0.8977 | 0.1296 | 0.9404 | 0.1099 | 0.5910 | 0.4725 |
| 23 | Deanery | Office | 0.5338 | 0.3033 | 0.9983 | 0.0059 | 0.8461 | 0.3060 |
| 24 | Learning Center | Computer room | 0.6682 | 0.2627 | 0.9974 | 0.0075 | 0.8156 | 0.3176 |
| 25 | Faculty council | Office | 0.9141 | 0.1139 | 0.9962 | 0.0124 | 0.8456 | 0.3063 |
| CA | Institute for Inorganic Chemistry | Laboratories | - | - | - | - | - | - |

CA: control area, pd: probability of direction, Rope: region of practical equivalence
