# Supplementary material for: Exposure levels of animal allergens, endotoxin, and β-(1,3)-glucan on a university campus of veterinary medicine
Source: PLoS One. 2023 Jul 13;18(7):e0288522. doi: 10.1371/journal.pone.0288522 (PMC10343150; doi:10.1371/journal.pone.0288522)
Supplement: S2 Table — CA: control area, N: number of measurements, ND: number of non-detectable samples, GM: geometric mean, GSD: geometric standard deviation. (DOCX) [file pone.0288522.s003.docx]

| **Location** | | | | **Fel d 1 (ng/m²)** | | | | **Can f 1 (ng/m²)** | | | | **Equ c 1 (ng/m²)** | | | | **Bos d 2 (ng/m²)** | | | |
| --- | --- | --- | --- | --- | --- | --- | --- | --- | --- | --- | --- | --- | --- | --- | --- | --- | --- | --- | --- |
| **No** | **Building** | **Room** | **N** | **ND** | **GM** | **GSD** | **Range** | **ND** | **GM** | **GSD** | **Range** | **ND** | **GM** | **GSD** | **Range** | **ND** | **GM** | **GSD** | **Range** |
| 1 | Small animal clinic, Internal Medicine | Seminar room | 11 | 0 | **262** | **1.7** | **122-702** | 0 | **1058** | **1.8** | **351-3398** | 0 | **721** | **2.2** | **92.4-2096** | 1 | 31.8 | 2.7 | <LOD-363 |
| 2 | Small animal clinic, Surgery | Ultrasound room | 12 | 0 | **605** | **1.8** | **236-1784** | 0 | **3727** | **1.9** | **1150-17162** | 0 | **5010** | **4.1** | **427-23195** | 2 | 49.5 | 3.3 | <LOD-607 |
| 3 |  | Anesthesia room | 12 | 0 | **3054** | **2.1** | **830-9659** | 0 | **12284** | **1.8** | **5481-35157** | 0 | **2295** | **2.7** | **460-16438** | **2** | **54.6** | **3.9** | **<LOD-977** |
| 4 |  | X-ray room | 12 | 0 | **83.5** | **1.4** | **42.7-126** | 0 | **1055** | **1.6** | **566-2890** | 0 | **13302** | **4.3** | **248-53635** | **0** | **119** | **2.0** | **<LOD-384** |
| 5 |  | Inpatient ward | 8 | 1 | **32.5** | **5.4** | **<LOD-1194** | 0 | **413** | **2.6** | **87.6-1649** | 0 | **66.4** | **2.4** | **28.7-433** | 4 | 14.6 | 1.7 | <LOD-39.5 |
| 6 |  | Exhibit collection | 12 | 0 | 24.6 | 2.1 | 6.7-104 | 0 | **276** | **2.3** | **48.8-701** | 0 | **706** | **2.4** | **126-2691** | 2 | 29.4 | 2.0 | <LOD-82.4 |
| 7 | Equine Clinic, Surgery | Examination room | 9 | 0 | 28.0 | 2.0 | 10.8-117 | 0 | **98.0** | **1.6** | **44.5-198** | 0 | **1126461** | **2.0** | **429582-2680916** | 0 | **333** | **3.5** | **81.8-3194** |
| 8 | Equine Horses, Internal Medicine | Corridor | 12 | 1 | **32.3** | **2.2** | **<LOD-101** | 0 | **296** | **2.4** | **71.8-1116** | 0 | **30805** | **12.3** | **123-276657** | 1 | **117** | **2.8** | **<LOD-539** |
| 9 | Ruminant Clinic | Lecture hall | 12 | 5 | 16.9 | 4.1 | <LOD-237 | 1 | 45.7 | 3.6 | <LOD-269 | 0 | **1210** | **2.2** | **533-7303** | 0 | **718** | **3.1** | **126-5956** |
| 10 | Clinic for Obsterics, Gynecology and Andrology | Lecture hall | 12 | 5 | 18.7 | 3.6 | <LOD-96.2 | 1 | **110** | **3.4** | **<LOD-367** | 0 | **5263** | **5.5** | **462-68569** | 0 | **1927** | **4.5** | **326-61055** |
| 11 |  | Lockerroom | 10 | 1 | **95.5** | **4.0** | **<LOD-553** | 0 | **369** | **3.3** | **18.1-1073** | 0 | **21021** | **4.9** | **672-108627** | 0 | **15777** | **3.2** | **945-75459** |
| 12 | Surgery lecture hall | Foyer | 11 | 3 | **42.6** | **5.0** | **<LOD-372** | 0 | **328** | **5.5** | **17.2-2133** | 0 | **1753** | **6.9** | **31.6-27020** | 1 | **56.4** | **2.5** | **<LOD-237** |
| 13 | Institute of Anatomy, Histology and Embryology | Practice room | 12 | 6 | 8.1 | 1.9 | <LOD-43.1 | 5 | 15.9 | 3.5 | <LOD-129 | 0 | 73.5 | 4.0 | 8.0-411.2 | 5 | 20.7 | 2.2 | <LOD-68.2 |
| 14 |  | Microscopy room | 12 | 5 | 13.4 | 2.6 | <LOD-47.4 | 3 | 30.2 | 3.5 | <LOD-131 | 1 | 144 | 4.4 | <LOD-610 | 4 | 25.8 | 2.4 | <LOD-106 |
| 15 |  | Lecture hall | 10 | 3 | 26.3 | 3.3 | <LOD-93.3 | 2 | 45.8 | 4.6 | <LOD-241 | 0 | **323** | **4.2** | **24.8-466** | 4 | 25.1 | 2.5 | <LOD-84.0 |
| 16 |  | Lockerroom | 11 | 3 | **75.3** | **8.6** | **<LOD-3117** | 3 | **108** | **9.8** | **<LOD-1866** | 1 | **621** | **19.6** | **<LOD-20074** | 2 | 48.2 | 2.9 | <LOD-294 |
| 17 | Institute of Physiology and Biochemistry | Lecture hall | 12 | 5 | 17.5 | 3.4 | <LOD-172 | 2 | **81.7** | **6.5** | **<LOD-1768** | 0 | **245** | **5.1** | **20.8-1253** | 3 | 33.7 | 2.4 | <LOD-126 |
| 18 |  | Practice room | 12 | 4 | 13.6 | 2.3 | <LOD-52.4 | 2 | **67.9** | **4.8** | **<LOD-603** | 0 | **237** | **4.0** | **12.7-1373** | 1 | **72.4** | **2.8** | **<LOD-310** |
| 19 | Institute of Food Science | Practice room | 11 | 4 | 14.6 | 2.7 | <LOD-63.2 | 4 | 25.5 | 4.4 | <LOD-165 | 1 | 102 | 3.7 | <LOD-557 | 1 | 43.7 | 1.9 | <LOD-91.2 |
| 20 | Institute of Pathology | Practice room | 12 | 8 | 8.0 | 2.3 | <LOD-58.9 | 6 | 11.9 | 2.9 | <LOD-108 | 0 | 96.3 | 4.3 | 11.5-1220 | 1 | 49.0 | 2.6 | <LOD-220 |
| 21 | Institute of Hygiene and Infectious Diseases | Lecture hall | 12 | 6 | 11.0 | 2.4 | <LOD-34.5 | 2 | 23.0 | 2.9 | <LOD-115 | 3 | 92.3 | 6.4 | <LOD-650 | 4 | 35.1 | 4.1 | <LOD-1301 |
| 22 |  | Practice room | 12 | 3 | 17.0 | 2.7 | <LOD-95.9 | 3 | 31.3 | 3.6 | <LOD-132 | 0 | 172 | 5.1 | 7.9-1253 | 4 | 31.5 | 3.2 | <LOD-220 |
| 23 | Deanery | Examination office | 12 | 7 | 6.7 | 1.5 | <LOD-12.9 | 0 | **149** | **4.6** | **9.3-885** | 0 | 172 | 3.4 | 16.5-1415 | 5 | 23.5 | 2.4 | <LOD-101 |
| 24 | Learning Center | Computer room | 10 | 1 | 25.0 | 2.9 | <LOD-121 | 1 | 41.3 | 2.6 | <LOD-93.1 | 0 | **483** | **3.1** | **103-3531** | 1 | 44.7 | 2.5 | <LOD-285 |
| 25 | Faculty council | Office | 12 | 6 | 11.2 | 2.6 | <LOD-76.1 | 1 | **53.2** | **3.5** | **<LOD-217** | 0 | **262** | **2.0** | **82.4-938** | 1 | 32.1 | 1.9 | <LOD-84.0 |
| **CA** | Institute for Inorganic Chemistry | Laboratories | 32 | 14 | 13.2 | 2.8 | <LOD-140 | 7 | 22.0 | 2.8 | <LOD-240 | 5 | 75.3 | 4.7 | <LOD-792 | 8 | 23.3 | 1.9 | <LOD-73.9 |

**S2 Table.** **Animal allergen, endotoxin and β-(1,3)-glucan levels at different locations of the veterinary medicine campus (descriptive statistics)**

CA: control area, N: number of measurements, ND: number of non-detectable samples, GM: geometric mean, GSD: geometric standard deviation

**S2 Table (continued).** **Animal allergen, endotoxin and β-(1,3)-glucan levels at different locations of the veterinary medicine campus (descriptive statistics)**

| **Location** | | | | **Mus m 1 (ng/m²)** | | | | **Domestic mite (ng/m²)** | | | | **β-(1,3)-glucan (ng/m²)** | | | | **Endotoxin (EU/m²)** | | | |
| --- | --- | --- | --- | --- | --- | --- | --- | --- | --- | --- | --- | --- | --- | --- | --- | --- | --- | --- | --- |
| **No** | **Building** | **Room** | **N** | **ND** | **GM** | **GSD** | **Range** | **ND** | **GM** | **GSD** | **Range** | **ND** | **GM** | **GSD** | **Range** | **ND** | **GM** | **GSD** | **Range** |
| 1 | Small animal clinic, Internal Medicine | Seminar room | 11 | 10 | 5.4 | 1.3 | <LOD-12.2 | 0 | 72.1 | 1.6 | 36.8-140 | - | **3411** | **1.7** | **903-5476** | - | **356** | **2.1** | **122-1214** |
| 2 | Small animal clinic, Surgery | Ultrasound room | 12 | 7 | 8.0 | 2.3 | <LOD-76.8 | 0 | **142** | **1.7** | **40.2-261** | - | **7124** | **3.4** | **225-18471** | - | **1192** | **2.0** | **346-3810** |
| 3 |  | Anesthesia room | 12 | 10 | 5.6 | 1.3 | <LOD-10.8 | 0 | **198** | **1.9** | **71.8-839** | - | **6476** | **2.7** | **1324-36768** | - | **1333** | **1.8** | **652-3414** |
| 4 |  | X-ray room | 12 | 7 | 8.5 | 2.3 | <LOD-61.0 | 0 | **134** | **1.6** | **63.9-368** | - | **10171** | **6.0** | **420-200875** | - | **1912** | **2.0** | **713-8852** |
| 5 |  | Inpatient ward | 8 | 8 | 5.0 | 1.0 | <LOD-<LOD | 7 | 25.1 | 1.2 | <LOD-38.0 | - | **1868** | **3.0** | **433-12132** | - | 59.4 | 2.7 | 11.5-233 |
| 6 |  | Exhibit collection | 12 | 12 | 5.0 | 1.0 | <LOD-<LOD | 7 | 35.4 | 1.8 | <LOD-103 | - | **2460** | **4.8** | **107-43698** | - | **184** | **2.4** | **35.4-549** |
| 7 | Equine Clinic, Surgery | Examination room | 9 | 0 | 99.5 | 2.4 | 37.3-379 | 0 | **3053** | **2.2** | **651-7842** | - | **374988** | **1.6** | **141986- 612978** | - | **91699** | **1.6** | **53513- 232890** |
| 8 | Equine Horses, Internal Medicine | Corridor | 12 | 1 | 127 | 4.6 | <LOD-834 | 0 | **6426** | **3.6** | **211-27114** | - | **69477** | **6.6** | **2091-567728** | - | **117322** | **3.3** | **8153-726674** |
| 9 | Ruminant Clinic | Lecture hall | 12 | 10 | 5.5 | 1.2 | <LOD--9.3 | 4 | **142** | **7.6** | **<LOD-19693** | - | **3589** | **3.2** | **567-25382** | - | **659** | **3.0** | **138-5180** |
| 10 | Clinic for Obsterics, Gynecology and Andrology | Lecture hall | 12 | 7 | 8.6 | 2.3 | <LOD-58.3 | 1 | **238** | **3.3** | **<LOD-2430** | - | **3834** | **5.5** | **233-53387** | - | **780** | **4.9** | **81.3-9507** |
| 11 |  | Locker room | 10 | 4 | 15.8 | 3.8 | <LOD-148 | 1 | **673** | **3.8** | **<LOD-3608** | - | **12455** | **6.1** | **716-196713** | - | **4127** | **3.8** | **175-21237** |
| 12 | Surgery lecture hall | Foyer | 11 | 11 | 5.0 | 1.0 | <LOD-<LOD | 2 | **122** | **3.4** | **<LOD-729** | - | **2885** | **2.7** | **704-14374** | - | **255** | **3.5** | **18.2-999** |
| 13 | Institute of Anatomy, Histology and Embryology | Practice room | 12 | 11 | 5.3 | 1.2 | <LOD-9.3 | 9 | 32.4 | 1.9 | <LOD-187 | - | 445 | 3.6 | 43.9-2242 | - | 56.3 | 3.6 | 16.3-804 |
| 14 |  | Microscopy room | 12 | 11 | 5.5 | 1.4 | <LOD-16.5 | 7 | 65.4 | 3.8 | <LOD-805 | - | 1000 | 2.7 | 83.3-3135 | - | 40.4 | 2.0 | 14.4-220 |
| 15 |  | Lecture hall | 10 | 11 | 5.0 | 1.0 | <LOD-<LOD | 3 | 114 | 3.8 | <LOD-663 | - | 1153 | 2.3 | 302-3289 | - | 59.0 | 2.7 | 11.5-287 |
| 16 |  | Locker room | 11 | 8 | 5.7 | 1.2 | <LOD-8.6 | 4 | **188** | **7.0** | **<LOD-2737** | - | **2438** | **4.0** | **442-41611** | - | 58.5 | 6.0 | 2.9-1616 |
| 17 | Institute of Physiology and Biochemistry | Lecture hall | 12 | 11 | 5.8 | 1.6 | <LOD-27.3 | 4 | 97.7 | 4.0 | <LOD-844 | - | **1529** | **2.7** | **387-8075** | - | 115 | 3.9 | 12.5-693 |
| 18 |  | Practice room | 12 | 11 | 5.2 | 1.1 | <LOD-7.9 | 7 | 38.9 | 2.0 | <LOD-149 | - | **2086** | **5.9** | **39.3-28752** | - | **159** | **2.8** | **22.3-602** |
| 19 | Institute of Food Science | Practice room | 11 | 10 | 5.2 | 1.1 | <LOD-7.9 | 8 | 32.6 | 1.8 | <LOD-135 | - | **1629** | **3.2** | **334-15892** | - | 49.1 | 3.1 | 5.7-180 |
| 20 | Institute of Pathology | Practice room | 12 | 11 | 5.2 | 1.1 | <LOD-7.9 | 7 | 74.1 | 9.5 | <LOD-51658 | - | **6827** | **4.7** | **317-83046** | - | **286** | **9.1** | **39.2-33449** |
| 21 | Institute of Hygiene and Infectious Diseases | Lecture hall | 12 | 11 | 5.0 | 1.0 | <LOD-<LOD | 7 | 35.5 | 1.7 | <LOD-82.5 | - | 688 | 2.8 | 49.8-4287 | - | 31.5 | 2.6 | 3.8-177 |
| 22 |  | Practice room | 12 | 12 | 5.0 | 1.0 | <LOD-<LOD | 9 | 34.9 | 2.1 | <LOD-164 | - | 1013 | 1.7 | 402-2184 | - | 42.7 | 2.3 | 13.4-168 |
| 23 | Deanery | Examination office | 12 | 12 | 5.0 | 1.0 | <LOD-<LOD | 6 | 42.7 | 2.3 | <LOD-372 | - | **1813** | **2.1** | **819-13243** | - | 67.3 | 2.3 | 13.4-174 |
| 24 | Learning Center | Computer room | 10 | 9 | 5.3 | 1.2 | <LOD-7.9 | 4 | 51.1 | 2.3 | <LOD-202 | - | **1886** | **2.4** | **792-7621** | - | 66.3 | 2.2 | 19.1-210 |
| 25 | Faculty council | Office | 12 | 11 | 5.2 | 1.1 | <LOD-7.9 | 9 | 33.5 | 2.1 | <LOD-249 | - | **1616** | **2.4** | **402-6880** | - | 67.2 | 2.5 | 14.4-385 |
| **CA** | Institut for Inorganic Chemistry | Laboratories | 32 | 32 | 5.0 | 1.0 | <LOD-<LOD | 17 | 48.4 | 2.5 | <LOD-467 | - | 530 | 5.1 | 19.3-11021 | - | 46.4 | 1.9 | 17.2-489 |

CA: control area, N: number of measurements, ND: number of non-detectable samples, GM: geometric mean, GSD: geometric standard deviation
