# Supplementary material for: Exposure levels of animal allergens, endotoxin, and β-(1,3)-glucan on a university campus of veterinary medicine
Source: PLoS One. 2023 Jul 13;18(7):e0288522. doi: 10.1371/journal.pone.0288522 (PMC10343150; doi:10.1371/journal.pone.0288522)
Supplement: S3 Table — pd: probability of direction, Rope: region of practical equivalence. (DOCX) [file pone.0288522.s004.docx]

**S3 Table.** **Results of Bayesian hypothesis tests - comparison of seasonal and annual variations of animal allergen, endotoxin and β-(1,3)-glucan levels**

| **Month and year** | **Fel d 1 (ng/m²)** | | **Can f 1 (ng/m²)** | | **Equ c 1 (ng/m²)** | | **Bos d 2 (ng/m²)** | |
| --- | --- | --- | --- | --- | --- | --- | --- | --- |
|  | **pd** | **ROPE** | **pd** | **ROPE** | **pd** | **ROPE** | **pd** | **ROPE** |
| Feb vs May | 0.8069 | 0.5097 | 0.6140 | 0.7516 | >0.9999 | 0.0004 | 0.9782 | 0.1538 |
| Feb vs Aug | >0.9999 | 0.0001 | >0.9999 | <0.0001 | 0.9362 | 0.3306 | 0.6973 | 0.6202 |
| Feb vs Nov | 0.9292 | 0.2943 | >0.9999 | 0.0020 | 0.9932 | 0.0857 | 0.7519 | 0.5743 |
| May vs Aug | >0.9999 | <0.0001 | >0.9999 | <0.0001 | >0.9999 | <0.0001 | 0.9341 | 0.3030 |
| May vs Nov | 0.7353 | 0.5826 | 0.9999 | 0.0049 | 0.9760 | 0.1870 | 0.9088 | 0.3623 |
| Aug vs Nov | >0.9999 | <0.0001 | >0.9999 | <0.0001 | >0.9999 | 0.0020 | 0.5664 | 0.6753 |
| 2014 vs 2015 | 0.7249 | 0.6520 | 0.5513 | 0.8318 | 0.8281 | 0.6176 | 0.9998 | 0.0091 |
| 2014 vs 2016 | 0.7018 | 0.6638 | 0.6978 | 0.7750 | 0.9104 | 0.4604 | 0.8551 | 0.5140 |
| 2015 vs 2016 | 0.5243 | 0.7208 | 0.7396 | 0.7421 | 0.6592 | 0.7578 | 0.9920 | 0.1032 |

| **Month and year** | **Domestic mite (ng/m^2^)** | | **β-(1,3)-glucan (ng/m²)** | | **Endotoxin (EU/m^2^)** | |
| --- | --- | --- | --- | --- | --- | --- |
|  | **pd** | **ROPE** | **pd** | **ROPE** | **pd** | **ROPE** |
| Feb vs May | 0.9513 | 0.1894 | 0.9993 | 0.0124 | 0.9525 | 0.3474 |
| Feb vs Aug | 0.5896 | 0.5474 | 0.9986 | 0.0236 | 0.6608 | 0.7554 |
| Feb vs Nov | 0.9970 | 0.0239 | 0.9553 | 0.2262 | 0.8247 | 0.6133 |
| May vs Aug | 0.9227 | 0.2554 | 0.5955 | 0.6629 | 0.9816 | 0.2069 |
| May vs Nov | 0.8828 | 0.3356 | >0.9999 | 0.0001 | 0.7631 | 0.6866 |
| Aug vs Nov | 0.9950 | 0.0368 | >0.9999 | 0.0002 | 0.9126 | 0.4594 |
| 2014 vs 2015 | 0.5956 | 0.6374 | 0.5152 | 0.7515 | 0.9971 | 0.1023 |
| 2014 vs 2016 | 0.8307 | 0.4529 | >0.9999 | <0.0001 | 0.9659 | 0.3556 |
| 2015 vs 2016 | 0.7663 | 0.5268 | >0.9999 | <0.0001 | 0.8085 | 0.7105 |

pd: probability of direction, Rope: region of practical equivalence
