# Supplementary figures and images for: Exposure levels of animal allergens, endotoxin, and β-(1,3)-glucan on a university campus of veterinary medicine
Source: PLoS One. 2023 Jul 13;18(7):e0288522. doi: 10.1371/journal.pone.0288522 (PMC10343150; doi:10.1371/journal.pone.0288522)

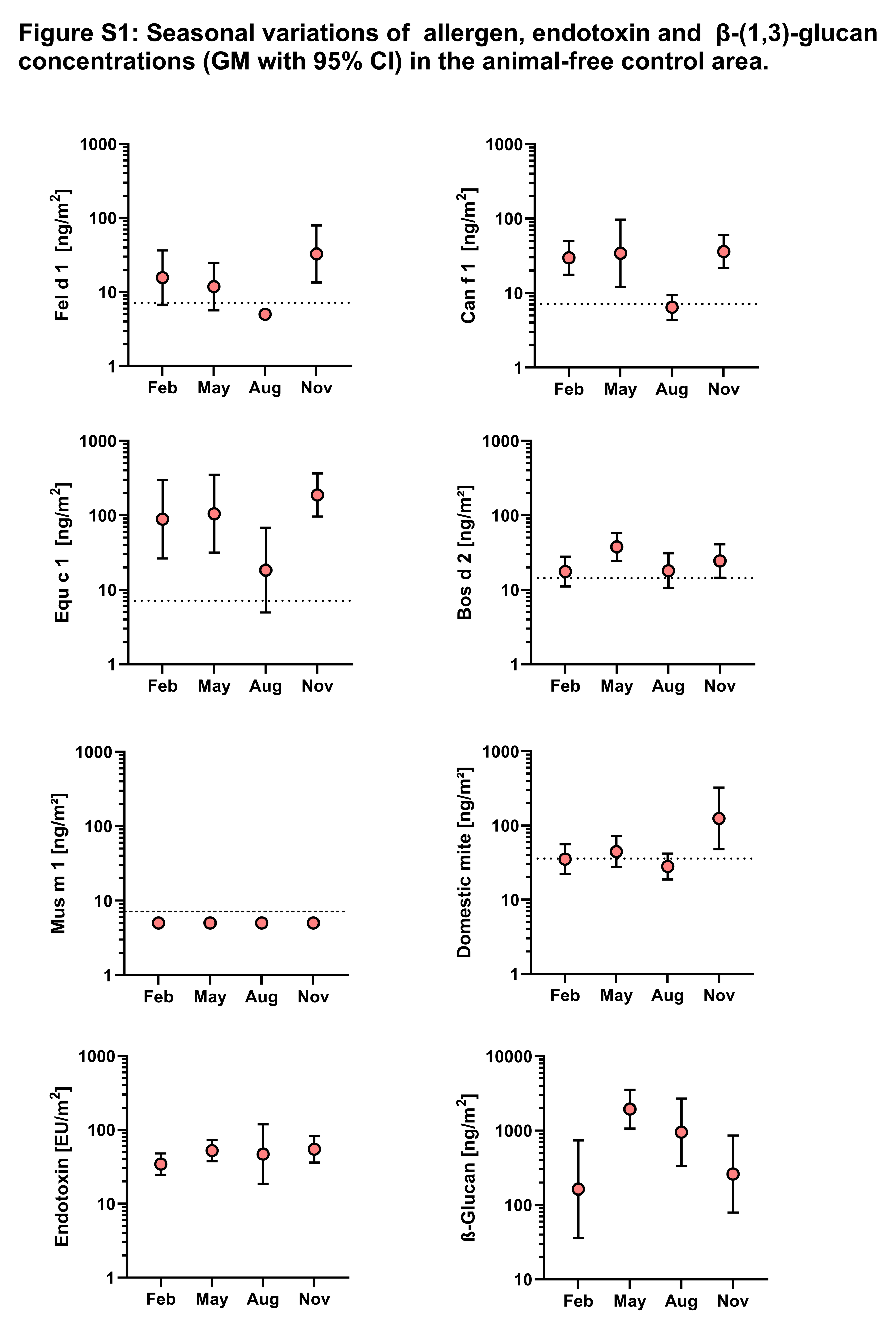

Supplement: S1 Fig — (TIF) [file pone.0288522.s005.tif]
